# Silvery fullerene in Ag_102_ nanosaucer

**DOI:** 10.1093/nsr/nwae192

**Published:** 2024-06-06

**Authors:** Zhi Wang, Yuchen Wang, Chengkai Zhang, Yan-Jie Zhu, Ke-Peng Song, Christine M Aikens, Chen-Ho Tung, Di Sun

**Affiliations:** School of Chemistry and Chemical Engineering, Shandong University, Ji'nan 250100, China; Department of Chemistry, Kansas State University, Manhattan 66506, USA; School of Chemistry and Chemical Engineering, Shandong University, Ji'nan 250100, China; School of Chemistry and Chemical Engineering, Shandong University, Ji'nan 250100, China; School of Chemistry and Chemical Engineering, Shandong University, Ji'nan 250100, China; Department of Chemistry, Kansas State University, Manhattan 66506, USA; School of Chemistry and Chemical Engineering, Shandong University, Ji'nan 250100, China; School of Chemistry and Chemical Engineering, Shandong University, Ji'nan 250100, China

**Keywords:** silvery fullerene, **Ag102** nanosaucer, cyclic anionic passivation layer, photothermal conversion, DFT calculation

## Abstract

Despite the discovery of a series of fullerenes and a handful of noncarbon clusters with the typical topology of *I*_h_-C_60_, the smallest fullerene with a large degree of curvature, C_20_, and its other-element counterparts are difficult to isolate experimentally. In coinage metal nanoclusters (NCs), the first all-gold fullerene, Au_32_, was discovered after a long-lasting pursuit, but the isolation of similar silvery fullerene structures is still challenging. Herein, we report a flying saucer-shaped 102-nuclei silver NC (**Ag102**) with a silvery fullerene kernel of Ag_32_, which is embraced by a robust cyclic anionic passivation layer of (KPO_4_)_10_. This Ag_32_ kernel can be viewed as a non-centered icosahedron Ag_12_ encaged into a dodecahedron Ag_20_, forming the silvery fullerene of Ag_12_@Ag_20_. The anionic layer (KPO_4_)_10_ is located at the interlayer between the Ag_32_ kernel and Ag_70_ shell, passivating the Ag_32_ silvery fullerene and templating the Ag_70_ shell. The *^t^*BuPhS^−^ and CF_3_COO^−^ ligands on the silver shell show a regioselective arrangement with the 60 *^t^*BuPhS^−^ ligands as expanders covering the upper and lower of the flying saucer and 10 CF_3_COO^−^ as terminators neatly encircling the edges of the structure. In addition, **Ag102** shows excellent photothermal conversion efficiency (*η*) from the visible to near-infrared region (*η* = 67.1% ± 0.9% at 450 nm, 60.9% ± 0.9% at 660 nm and 50.2% ± 0.5% at 808 nm), rendering it a promising material for photothermal converters and potential application in remote laser ignition. This work not only captures silver kernels with the topology of the smallest fullerene C_20_, but also provides a pathway for incorporating alkali metal (M) into coinage metal NCs via M-oxoanions.

## Teaser

The “silvery fullerene” of Ag32, trapped in Ag_102_ nanosaucer and passivated by a cyclic anionic layer of (KPO4)20, has been synthesized and demonstrates significant potential as a photothermal conversion material for remote laser ignition.

## INTRODUCTION

The persistent fascination with coinage metal nanoclusters (NCs) arises from their remarkable structural diversity involving the distinctive metallophilicity and their promising applications in catalysis, luminescence, biomedicine, as well as chemical sensing [1–9]. Understanding the precise structure is of utmost importance as it holds the key to advancing our knowledge about the evolution from a discrete metal atom to a solid state [10–16]. In 2004, Johansson, Gong, *et al.* made a noteworthy prediction, suggesting that the most stable structure of the Au_32_ cage would be a hollow structure with icosahedral (*I*_h_) symmetry and a diameter of ∼0.9 nm [17,18]. The synthesis and total structure determination of golden fullerene were carried out by Wang and Schnepf’s groups in 2019, employing the direct reduction of the Au precursor by NaBH_4_ and X-ray crystallography characterization [19,20]. This marked the emergence of the first all-gold fullerene species. In light of the successful elucidation of the golden fullerene, can the silvery fullerene with a similar topology be achieved? A fullerene-like silver NC of Ag_13_@Ag_20_ was previously reported, but the inner kernel is a centered-icosahedral Ag@Ag_12_ solid rather than a hollow cage and the outer dodecahedral shell is distorted, which deviates from the ideal fullerene topology [21]. Over the past decades, a popular synthetic strategy for silver NCs was the bottom-up approach involving the chemical reduction of silver-ligand complexes in solution or the introduction of directing agents to facilitate the aggregation of silver ions [22,23]. Significant progress has been achieved for organic ligand-protected silver NCs such as the largest silver cage, Ag_180_, and the largest silver nanoparticle, Ag_374_ [24,25], both of which were characterized by X-ray crystallography. Despite significant efforts dedicated to synthesizing and elucidating the structure of silver NCs, the total structural determination of silvery fullerene Ag_32_ remains pending.

Recently, *N,N*-dimethylformamide (DMF) has demonstrated efficacy as a mild reductive agent, slowing down the reductive rate of silver ions to atoms, then decelerating the aggregation rate of silver atoms into nanoparticles. This, in turn, enables oxoanions to passivate the highly active silver aggregates. As a matter of fact, nanoscale icosahedral silver nanocrystals can be produced by the reduction of AgNO_3_/PVP in DMF [26]. If downsizing the icosahedral silver nanocrystal to the molecular level, it yields the dual polyhedron Ag_12_ of Ag_20_ with minimal fullerene topology, promising the potential to obtain the structure of silvery fullerene Ag_12_@Ag_20_ by using DMF as the reductive agent. Our group has successfully isolated a series of silver NCs that encapsulated different silver kernels, such as [Ag_6_@(CrO_4_)_8_@Ag_52_], [Ag_10_@(Mo_7_O_26_)_2_@Ag_70_], [Ag_6_@(MoO_4_)_7_@Ag_56_] and [Ag_13_@Ag_76_S_16_(*p*-NH_2_-PhAsO_3_)_4_], which display the excellent abilities of anionic templates in passivating the inner silver kernel and supporting the outer silver shell [27–30]. However, the trapping of silvery fullerene structures has not yet been observed, which may be related to the incompatibility of the silver kernel with the oxoanionic passivation layer. Considering that both coinage and alkali metal (M) atoms have a valence shell *s*^1^ electronic configuration [31], it is plausible that the M could be incorporated into the silver NCs to support the overall structure [32–34]. However, alkali and coinage metal atoms differ greatly in terms of atomic radius size and standard reduction potential [35], presenting substantial challenges for incorporating M into silver NCs. Zheng *et al.* reported the first case regarding the incorporation of M into an alkynyl-stabilized coinage-metal NC, a body-centered cubic (bcc) structure of [Au_7_Ag_8_(C≡C*^t^*Bu)_12_]^+^ and the substitution chemistry of that cluster was well studied by electrospray ionization mass spectrometry (ESI-MS) and density functional theory (DFT) calculations, but the single-crystal structure involving the M incorporation has not been available [31]. We envision that oxygenphilic M may be incorporated into the silver NCs through M-O bonding to form an M-oxoanions passivation layer to capture the silvery fullerene. Based on the above considerations, the isolation of the silvery fullerene by M-oxoanions is significant but full of challenges.

To achieve this objective, with the endeavor to introduce rarely utilized KH_2_PO_4_ species in the assembly of silver NCs, we successfully isolated a giant 102-nuclei silver NC (**Ag102**) with an unprecedented silvery fullerene kernel. In a shape resembling a flying saucer, **Ag102** has the following features: (i) a robust anionic cyclic passivation layer of (KPO_4_)_10_ trapping a silvery fullerene kernel of Ag_32_; (ii) the first Ag-K bimetallic NC at atomic precision level; (iii) regiospecific distribution of *^t^*BuPhS^−^ and CF_3_COO^−^ ligands on the silver shell.

## RESULTS

**Scheme 1.**
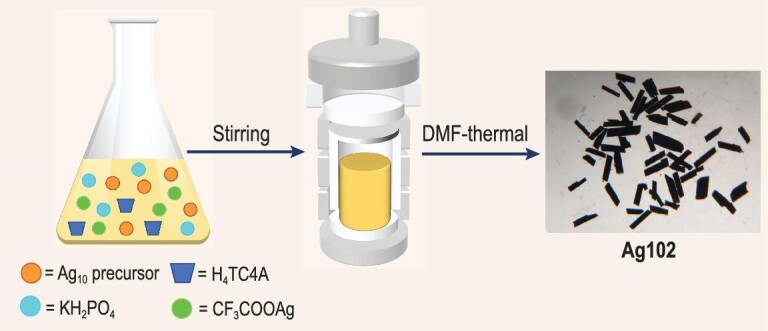
Synthetic route for **Ag102**. Ag_10_ precursor = {(HNEt_3_)_2_[Ag_10_(*^t^*BuPhS)_12_]}*_n_*, H_4_TC4A = *p*-tert-butylthiacalix[4]arene.

The preparative route of **Ag102** involved the direct reduction of a mixture of {(HNEt_3_)_2_[Ag_10_(*^t^*BuPhS)_12_]}*_n_, p*-tert-butylthiacalix[4]arene (H_4_TC4A), CF_3_COOAg and KH_2_PO_4_ in the aprotic solvent DMF. Black rod-like crystals suitable for X-ray diffraction were obtained after DMF-thermal reaction (Scheme 1). Detailed synthetic procedures are provided in the Supplementary Information (SI). In the synthetic process, DMF serves a dual role both as a solvent to facilitate the dissolution of the above-mentioned reactants, and as a mild reducing agent to promote the reduction of Ag^+^, followed by the formation of embryonic silver kernels in a controllable manner. To the best of our knowledge, the silvery fullerene of Ag_32_ captured in **Ag102** is the largest subvalent silver kernel with DMF as the mild reducing agent (Table S1). Although the H_4_TC4A ligand is not involved in the final structure of **Ag102**, the black precipitate was exclusively obtained in the absence of it, indicating its important role in the formation of **Ag102**. The possible reason was that H_4_TC4A first reacted with Ag(I) to form the intermediate Ag-TC4A complex, which can slow down the release rate of Ag(I) ions to further diminish the reduction kinetics of DMF, finally forming a medium-sized subvalent silver kernel.

### Crystal structure of Ag_102_

**Figure 1.**
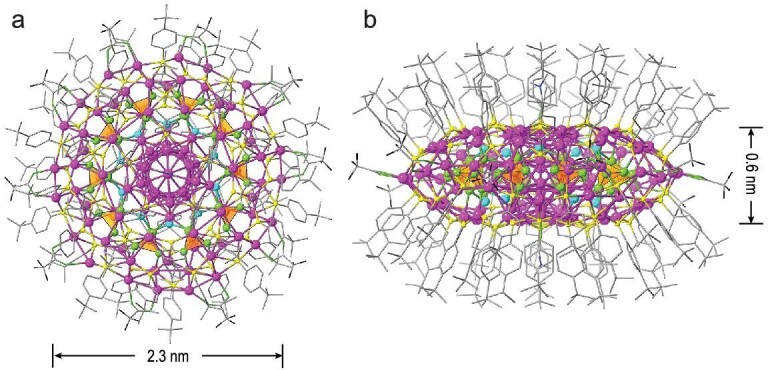
Structure of **Ag102**: Top (a) and side (b) views. Color labels: purple, Ag; cyan, K; black, F; orange, P; blue, N; yellow, S; gray, C; green, O. The orange tetrahedra represent PO_4_^3−^.

Single-crystal X-ray diffraction (SCXRD) analysis revealed that **Ag102** crystallized in a monoclinic space group *I*2/*m* with the formula of [Ag_12_@Ag_20_@(KPO_4_)_10_@Ag_70_(*^t^*BuPhS)_60_(CF_3_COO)_10_(DMF)_2_]. The entire **Ag102** looks like a flying saucer and exhibits a pseudo–5-fold symmetry (Fig. 1). The equatorial diameter and axial thickness of the silver skeleton are 2.3 and 0.6 nm, respectively. The high-angle annular dark-field scanning transmission electron microscopy (HAADF-STEM) images reveal that the average size of the nanoparticles is ∼2.2 nm (Fig. S1), in agreement with the equatorial diameter (2.3 nm) of **Ag102** determined by SCXRD (Fig. 1a). There are two Ag_102_ molecules in a unit cell, one at the apex and the other at the center (Fig. S2). In detail, the structure of **Ag102** features a three-layered structure with an Ag_12_@Ag_20_ silvery fullerene as the subvalent kernel, a (KPO_4_)_10_ anionic layer, and an Ag_70_ shell from inner to outer. The Ag_70_ shell is protected by the binary organic ligands of *^t^*BuPhS^−^ and CF_3_COO^−^.

**Figure 2.**
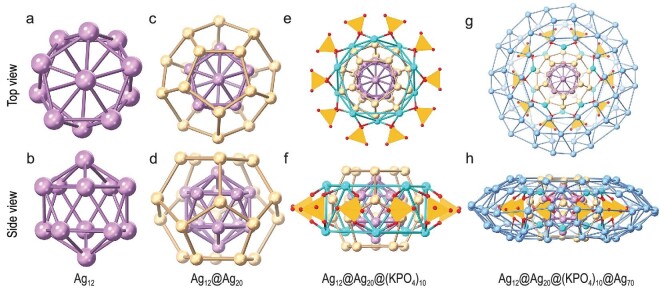
Top and side views of the structure dissection of **Ag102**. (a, b) The innermost icosahedron Ag_12_ (lilac); (c, d) the dodecahedron Ag_20_ (golden) trapping inner icosahedron Ag_12_; (e, f) the (KPO_4_)_10_ anion shell embracing Ag_12_@Ag_20_ kernel (cyan, K; red, O; orange, P); (g, h) the Ag_70_ shell (wathet) encaging inner Ag_12_@Ag_20_@(KPO_4_)_10_.

For a more elaborate anatomy of the structure, we start with the innermost part, Ag_12_. As portrayed in Fig. 2a and b, the Ag_12_ is a hollow icosahedron. In contrast to the most observed non-hollow icosahedral Ag_13_ kernel, the hollow icosahedral Ag_12_ kernel in **Ag102** is also an important nano-building block of silver NCs, as observed in Ag_44_(SR)_12_, Ag_50_(dppm)_6_(SR)_30_, etc [36–38]. The Ag···Ag distance in Ag_12_ falls in the range of 2.748–2.847 Å (average: 2.808 Å). Subsequently, an Ag_20_ dodecahedron completely embraces the Ag_12_ hollow icosahedral cage forming a silvery fullerene kernel (Ag_12_@Ag_20_). In this configuration, the icosahedron and its dual (the dodecahedron) with *I_h_* symmetry are thus compatible, and each silver atom of Ag_12_ is directed toward the center of the pentagon in the Ag_20_ dodecahedron (Fig. 2c, d). The Ag···Ag distances between Ag_12_ and Ag_20_ are in the range of 2.760–2.986 Å (average: 2.847 Å; Fig. S3). The 30 Ag···Ag edge lengths in the Ag_20_ fall in the range of 3.005–3.481 Å, of which 10 longer Ag···Ag edges (two in 3.349 Å, four in 3.359 Å and four in 3.481 Å) were elongated by the coordination of *^t^*BuPhS^−^. The size of the Ag_20_ is ∼7.1 Å, identical in size to its famous all-carbon fullerene cousin, C_60_, and slightly shorter than the size of [K@Au_12_Sb_20_]^5−^ (Fig. S4) [39–41]. The anionic passivation layer of (KPO_4_)_10_ situated at the waist of Ag_12_@Ag_20_ plays a dual role of passivating the inner Ag_12_@Ag_20_ kernel and supporting the outer Ag_70_ shell. There are no other organic ligands except two DMF molecules that penetrated the Ag_20_ top and bottom pentagons and anchored on the top and bottom silver atoms of the Ag_12_ hollow cage with Ag-O_DMF_ bond distances of 2.334 Å. Each of the remaining 10 pentagon faces of the Ag_20_ dodecahedron is coated with a K^+^ ion, resulting in the formation of a K_10_ pentagonal antiprism, which can be derived from the regular icosahedron by cutting off two opposite vertices (Figs S5, S6). Each PO_4_^3−^ rides on the K-K edge of the waist of the K_10_ pentagonal antiprism and ligates two K atoms via two of the four O atoms to form the (KPO_4_)_10_ cyclic anionic layer (Fig. 2e, f). One PO_4_^3−^ can attach six silver atoms, two of the Ag_20_ dodecahedron and four of the outer Ag_70_ shell, giving the coordination pattern of *μ*_8_-*κ*^3^:*κ*^3^:*κ*^1^:*κ*^1^ (Ag–O: 2.254–2.504 Å and K–O: 2.224–2.352 Å) (Fig. 2g, h).

**Figure 3.**
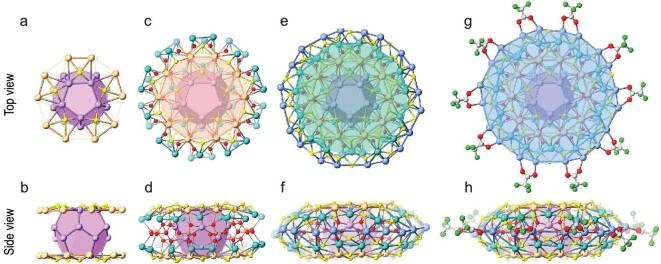
Top and side views of the four hierarchies surrounding the Ag_32_ kernel in **Ag102**. (a, b) The first hierarchy Ag_20_S_10_ (golden, Ag); (c, d) the second hierarchy Ag_30_S_20_ (dark green, Ag); (e, f) the third hierarchy Ag_20_S_30_ (wathet, Ag); (g, h) the fourth hierarchy 10 CF_3_COO^−^. Color labels: cyan, K; green, F; orange, P; yellow, S; gray, C; red, O.

From the polygon view, the outer conch-shaped Ag_70_ shell is composed of silver trigons and tetragons, revealing an intriguing interfacial binding profile, with *^t^*BuPhS^−^ acting as expanding ligands covered on the Ag_4_ tetragons and Ag_3_ trigons at the up and down of the conch, and CF_3_COO^−^ as terminating ligands which seamed the periphery, respectively (Figs S7, S8). The surface protection patterns can be divided into four hierarchies from the apex to the equator: Ag_20_S_10_, Ag_30_S_20_, Ag_20_S_30_ and terminating ligands (10 CF_3_COO^−^). To clearly illustrate the interfacial binding profiles and silver arrangements, we describe the structure of **Ag102** from the kernel outward. The first hierarchy contains two Ag_10_ rings at the upper and lower faces and 10 *^t^*BuPhS^−^ coordinated to silver atoms via the *μ*_4_ coordination pattern (Fig. 3a, b). Namely, the Ag_10_ ring of the first hierarchy ligates with the Ag_20_ dodecahedron of the Ag_32_ kernel to form five vertex-sharing Ag_4_ tetragons. The second hierarchy is composed of two Ag_15_ rings and 20 *^t^*BuPhS^−^ ligands, with the *^t^*BuPhS^−^ ligands connecting the inner Ag_10_ and Ag_15_ rings in *μ*_3_ and *μ*_4_ coordination modes (Fig. 3c, d). The cyclic (KPO_4_)_10_ acts as not only the anion passivation layer to impede further growth of the inner Ag_32_ kernel but also the anionic template to support the outer Ag_70_ shell (Fig. S9). As the outermost silver layer, the third hierarchy consists of an Ag_20_ ring at the equator and 30 *^t^*BuPhS^−^ ligands arranged in alternating *μ*_3_ and *μ*_4_ coordination modes (Fig. 3e, f). Interestingly, the *^t^*BuPhS^−^ ligands coordinate with the distorted Ag_4_ square, which can be seen as a transition from the conventional S-Ag_4_ square to the S-Ag_3_ triangle. Finally, the fourth hierarchy consists of 10 CF_3_COO^−^ in *μ*_2_-*κ*^1^:*κ*^1^ coordination pattern as terminator ligands coordinated on the periphery of saucer-shaped **Ag102** (Fig. 3g, h).

**Figure 4.**
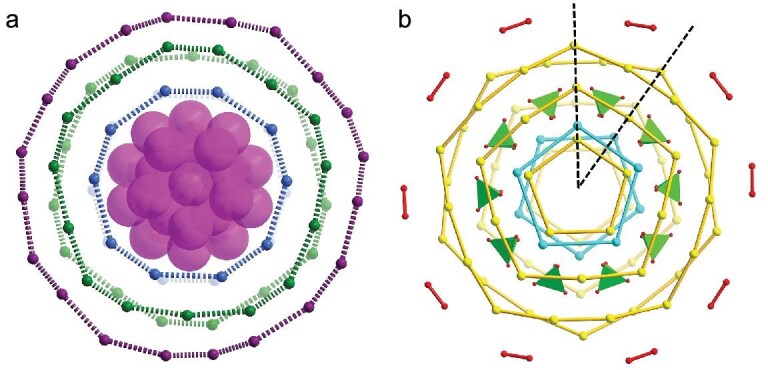
(a) Layer-by-layer structure of the silver atoms in **Ag102**. Ag_32_ kernel is represented in purple space-filling mode and three layers are highlighted individually in different colors (blue, green, modena). (b) The distribution of 60 *^t^*BuPhS^−^ ligands (yellow), 10 K^+^ (cyan), 10 PO_4_^3−^ (green tetrahedra) and 10 CF_3_COO^−^ (red).

Even more strikingly, Ag_12_@Ag_20_ kernel, (KPO_4_)_10_ anion layer, and each of the aforementioned hierarchies exhibit high symmetry. The 102 Ag atoms can be divided into two categories: an *I*_h_ symmetric silvery fullerene Ag_12_@Ag_20_ kernel which contains the concentric icosahedral Ag_12_ as well as dodecahedral Ag_20_ and an Ag_70_ shell in a layer-by-layer arrangement (Fig. 4a). After passivation by (KPO_4_)_10_, the symmetry transitions from *I_h_* symmetry in Ag_12_@Ag_20_ to *D_5d_* symmetry in Ag_12_@Ag_20_@(KPO_4_)_10_. The peripheral 70 Ag atoms are distributed in concentric circles, with the two Ag_10_ rings (blue) in the first layer, the two Ag_15_ rings (green) in the second layer and a large silver ring of Ag_20_ (modena) in the outermost layer. Each Ag_10_ ring and Ag_15_ ring are parallel to each other but rotated by 36.5^o^ (Ag2-Ag20-Ag20'-Ag14) and 36.8^o^ (Ag15-Ag20-Ag20'-Ag3), respectively (Fig. S10). The adjacent silver rings and the inner kernel are connected by *^t^*BuPhS^−^ ligands. Moreover, the *^t^*BuPhS^−^ ligands on each side of the top and bottom of the **Ag102** surface together with K^+^ ions show a total of eight pentagonal patterns (Fig. 4b). This may be related to the fact that they initially form the S_5_ and K_5_ pentagonal pattern by binding S to the Ag_5_ edge and capping K to the Ag_5_ face of the dodecahedron Ag_20_ via five Ag_4_S and Ag_5_K units, respectively (Figs 2e, f and 3a, b), reflecting good transitivity. Interestingly, each pentagon shows similar relative arrangements like Ag_10_ and Ag_15_ rings, with each parallel pentagon rotated by ∼36^o^ with respect to each other. Ten PO_4_^3−^ are arranged into a P_10_ decagon on the waist of **Ag102**. The arrangements of the silver atoms, K^+^ and the *^t^*BuPhS^−^ ligands are all like the staggered ferrocene, giving the whole structure a *D*_5d_ symmetry.

### Electrospray ionization mass spectrometry

Apart from SCXRD, the charge state and the solution behavior of **Ag102** were further investigated by electrospray ionization mass spectrometry (ESI-MS) [42–45]. In the positive-ion mode of ESI-MS for **Ag102** dissolved in the mixed solvent of CH_2_Cl_2_-CH_3_OH, three prominent sets of peaks are observed at *m/z* of 4000–8000 for +5 (**a**–**g**), +4 (**h**–**l**) and +3 (**m**–**q**) charge states, with +3 species being the most abundant (Figs S11–S14). After carefully comparing the experimental isotope distributions with the simulated envelopes, no parent ions of Ag_102_ were identified in either of them. The most abundant +3 charged species (**m**–**q**) can be assigned with the following formulae, where **Δ**=Ag_12_@Ag_20_@(KPO_4_)_10_: {**Δ**@Ag_65_(*^t^*BuPhS)_60_(CF_3_COO)_2_(H_2_O)}^3+^ (**m**); {**Δ**@Ag_66_(*^t^*BuPhS)_58_(CF_3_COO)_5_(solvents)}^3+^ (solvents = H_2_O for **n**; 2CH_3_OH for **o**); {**Δ**@Ag_66_(*^t^*BuPhS)_60_(CF_3_COO)_3_(solvents)}^3+^ (solvents = CH_3_OH and 2H_2_O for **p**; CH_3_OH and 2 DMF for **q**) (Fig. S12). The remaining two isotope groups are also assigned and their formulae are summarized in tabular form (Figs S13, S14). These fragment species of Ag_102_ mainly result from the dissociation of the organic components, such as CF_3_COO^−^ and *^t^*BuPhS^−^ ligands, as well as exterior Ag atoms, while the inner [Ag_12_@Ag_20_@(KPO_4_)_10_] remains intact. From these assigned formulae, the number of free electrons for **Ag102** was deduced to be 12, and its electronic structure is an incomplete shell configuration 1S^2^1P^6^1D^4^.

### Density functional theory calculation

**Figure 5.**
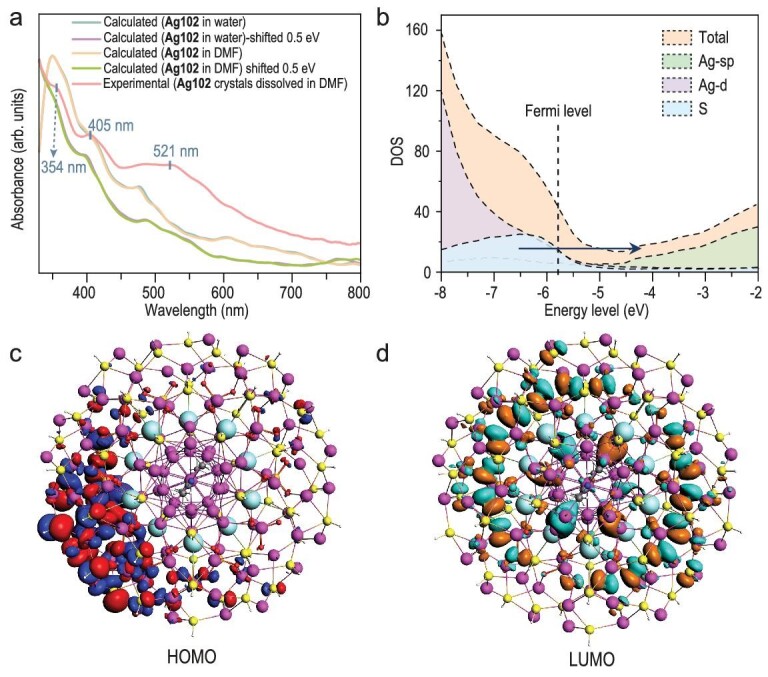
(a) Experimental and calculated UV-Vis absorption spectra of **Ag102**, and the experimental UV-Vis absorption spectrum of **Ag102** recorded in DMF solution at room temperature. (b) DOS around the Fermi level of the **Ag102**; the arrow indicates the dominant transitions for the three excitation peaks. (c) HOMO and (d) LUMO for the **Ag102** system (isovalue = 0.01 au). Color labels: purple, Ag; cyan, K; orange, P; blue, N; yellow, S; gray, C; red, O; white, H.

The room temperature UV-Vis absorption spectrum of **Ag102** crystals dissolved in DMF exhibits three absorption bands at 354 nm, 405 nm and 521 nm within the 330 to 800 nm range (Fig. 5a). In order to unveil the electronic structure of **Ag102**, density functional theory (DFT) calculations were performed (Fig. S15). The calculated time-dependent DFT plus tight binding (TDDFT+TB) spectrum shows good agreement with the experimental spectrum when a constant shift of 0.5 eV is applied to the theoretical spectrum. The shifted calculated optical spectrum (Fig. 5a) exhibits several peaks, including peaks at 355 nm (peak 1), 398 nm (peak 2) and 488 nm (peak 3), whereas the unshifted spectrum exhibits peaks at ∼400 nm (peak 1), 480 nm (peak 2) and 606 nm (peak 3), respectively. The spectra look almost the same whether water or DMF is used as an implicit solvent in the calculations. GGA functionals often underestimate the energies of excitations, and a shift of 0.5 eV is reasonable to correct the excitation energy results compared with experiments. The DOS (density-of-states) around the Fermi level is shown in Fig. 5b. For peak 1, peak 2 and peak 3, the major electronic transitions are dominated by the sulfur atomic orbitals to the *s* and *p* orbitals of silver atoms. Each broad excitation peak is composed of many different electronic excited states. These three peaks primarily arise from transitions between occupied orbitals that originate from sulfur atomic orbitals into unoccupied orbitals that primarily arise from *s* and *p* atomic orbitals on Ag. The highest occupied molecular orbital (HOMO) and lowest unoccupied molecular orbital (LUMO), which are representative of these two types of orbitals, are shown in Fig. 5c, d. Due to those orbital characters, the main electronic transitions occur with electron density moving from the outer shell to the inner shell and kernel of the system. The UV-Vis absorption spectrum of **Ag102** crystals dissolved in trichloromethane is shown in Fig. S16, and the spectrum shows one distinct peak at 478 nm within the same range.

### Photothermal conversion

**Figure 6.**
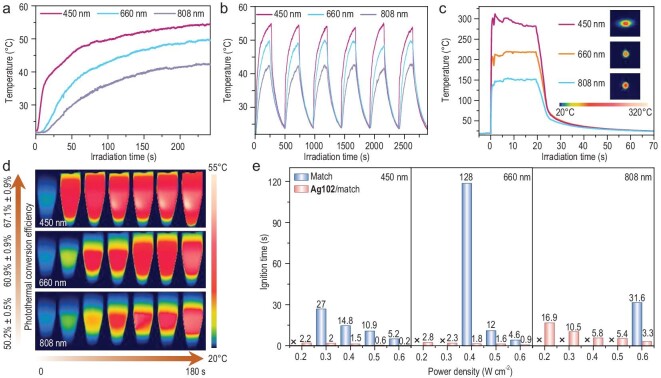
Photothermal conversion curves (a), photothermal heating and cooling cycles (b) and thermal images (d) of the CHCl_3_ solution of **Ag102** at the concentration of 100 µM under 450, 660 and 808 nm laser irradiation (0.3 W cm^−2^). (c) Photothermal conversion curves of **Ag102** in the solid state under 450, 660 and 808 nm laser irradiation (0.3 W cm^−2^). Insets: thermal images of **Ag102** at the highest temperature. (e) The comparison of the ignition time of match and **Ag102**/match at different laser power densities at wavelengths of 450, 660 and 808 nm, respectively. The numbers in the figure represent the ignition time (seconds), the cross means it cannot be ignited. The error bars in Fig. 6d were determined from the standard deviation of three parallel experiments.

The optical and photothermal conversion performance of **Ag102** was investigated under laser irradiation at the wavelengths of 450, 660 and 808 nm (0.3 W cm^−2^). The temperature of the CHCl_3_ solution of **Ag102** (100 μM) rapidly reaches the maximum temperature within 4 min and the corresponding maximal temperatures are 54.4, 49.6 and 42.3°C under 450, 660 and 808 nm laser irradiation, respectively (Fig. 6a, d). Moreover, at each time point, the temperature of the CHCl_3_ solution of **Ag102** under 450 nm irradiation was higher than the others, indicating that it has better photothermal conversion performance at 450 nm. The maximal temperature was almost unchanged in 6 cycles of photothermal heating and cooling processes within a time span of 48 min (Fig. 6b). A comparison of the UV-Vis spectra of the CHCl_3_ solution of **Ag102** before and after laser irradiation also confirmed its excellent photothermal stability (Fig. S16). The photothermal conversion efficiency (*η*) of the CHCl_3_ solution of **Ag102** was calculated to be 67.1% ± 0.9%, 60.9% ± 0.9% and 50.2% ± 0.5% at 450 nm, 660 nm and 808 nm laser irradiation, respectively (Figs S17–S19). The *η* of **Ag102** at 450 nm is comparable to the recently reported promising photothermal materials Au_44_MBA_26_-Cy7 (65.12%) and (AgCu)_31_ (67.6%) [46,47], and much higher than the previously reported Au_44_MBA_26_ (34.96%), Ag_16_ NCs (41.1%, 35.8%, 40.7% and 33.6%), silver nanocages (46.1%) and silver nanoparticles (36.8%), etc. (Fig. S20) [48–55].

**Ag102** exhibits a broad absorption spanning the UV and NIR regions in the solid state (Fig. S21) and is free of luminescence, suggesting that non-radiative migration is the main route of energy release, so we further investigated the photothermal conversion of **Ag102** in the solid state [56,57]. The temperature of **Ag102** in the solid state increased from 20 to 301°C in 0.9 s under 450 nm low-power laser irradiation (0.3 W cm^−2^). The temperature increase of **Ag102** was 206.1°C (208°C s^−1^) under 660 nm laser irradiation in solid state, while it was 138.5°C (133°C s^−1^) under 808 nm laser irradiation (Fig. 6c). Although the maximal temperatures of the other laser irradiation wavelengths are lower compared to 450 nm, it still has a fast temperature increase rate. In view of the broad absorption and photothermal conversion properties of **Ag102**, we contemplate its potential as a candidate for a remote laser ignition material. A match was used as a model for the remote laser ignition experiment. Prior to the laser ignition tests, we dispersed 2 mg **Ag102** in ethanol, applied it evenly to the match head, and then left it for ∼2 h to allow the ethanol to evaporate. The as-obtained sample is hereafter referred to as **Ag102**/match. The laser ignition threshold power and ignition time of match and **Ag102**/match were measured by changing the laser intensity at a fixed distance of 20 cm. The temperature evolution of the sample over time was recorded using a thermal imaging camera (range: 0–650°C). As shown in Fig. S22, match and **Ag102**/match were irradiated under three different wavelengths of laser (450, 660 and 808 nm) with the power densities of 0.2–0.6 W cm^−2^. Notably, under 450 nm laser irradiation, the match could not be ignited at 0.2 W cm^−2^, whereas **Ag102**/match was ignited within 2.2 s, and as the power density increased to 0.6 W cm^−2^, the ignition time of **Ag102**/match was further reduced to 0.2 s (Fig. 6e). When subjected to 660 nm laser irradiation, the match can be ignited until the power density increases to 0.4 W cm^−2^ with an ignition time of 128 s (Fig. 6e). In contrast, **Ag102**/match can be ignited within the range of 0.2 to 0.6 W cm^−2^, with an ignition time reduced to 1.8 s at 0.4 W cm^−2^. Furthermore, when exposed to 808 nm laser irradiation, the match can only be ignited when the power density increases to 0.6 W cm^−2^, necessitating 31.6 s for ignition. In the range of power densities between 0.2 and 0.6 W cm^−2^, **Ag102**/match can be ignited and the ignition time decreases from 16.9 to 3.3 s (Fig. 6e). Coating **Ag102** on the surface of the match shortened the ignition time and decreased the laser ignition threshold power. In addition, **Ag102**/match has the shortest ignition time under 450 nm laser irradiation, which is related to its strong absorption and better photothermal conversion performance at 450 nm. These results suggest that **Ag102** shows significant promise as a photo-responsive material for further applications in remote laser ignition, which is also very important for the development of laser initiators with low initiation energy in the future.

### Unraveling the electronic dynamics of Ag102

**Figure 7.**
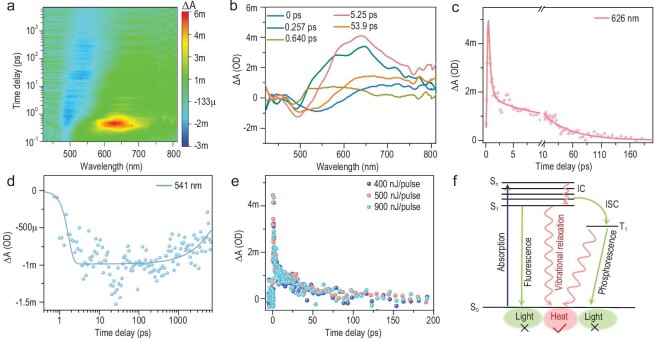
fs-TA characterization of the **Ag102**. (a) Transient absorption data map pumped at 400 nm. (b) The TA spectra of **Ag102** at different time delays. (c) Kinetic traces probed at 626 nm. (d) Kinetic traces at 541 nm and the corresponding fitting. (e) Excitation pulse energy dependent kinetics. (f) Simplified Jablonski diagram illustrating photophysical processes of **Ag102** at room temperature. IC: internal conversion, ISC: intersystem crossing.

Whether phonon-assisted non-radiative decay or surface plasmon resonance (SPR) dominates the pathway of photothermal conversion of silver clusters such as **Ag102** is an enduring question [58–60]. Herein, femtosecond transient absorption (fs-TA) measurements were performed to probe the electronic dynamics of **Ag102**. Upon excitation with a 400 nm laser pulse, rich electronic dynamics can be observed within the 7 ns time window, and an apparent ground-state bleaching (GSB) signal near 500 nm can be identified in the fs-TA spectrum, corresponding to the absorption band of **Ag102**, along with a net excited-state absorption (ESA) signal ∼626 nm in the visible range (Fig. 7a and b). Specifically, the analysis of dynamics traces of 626 nm ESA gives three decay components (*τ*_1_ = 383.3 fs, *τ*_2_ = 4.613 ps and *τ*_3_ = 48.67 ps) (Fig. 7c), in which the 383.3 fs component should be assigned to the internal conversion (IC) from *S*_n_ to *S*_1_ state [61,62], and subsequent picosecond component 4.613 ps may be attributed to structural relaxation within the large cluster. Combined with the long-lived GSB signal in the whole 7 ns time-window, this component may correspond to the relaxation of the excited state electrons from *T*_1_ to *S*_0_, indicating the existence of a triplet excited state (Fig. 7d). Owing to the small energy gap in **Ag102**, another picosecond component 48.67 ps could be the electron relaxation from *S*_1_ to the ground state (*S*_0_). Moreover, the excitation-pulse-energy-dependent kinetics were investigated using TA measurements (Fig. 7e), the ESA signals were selected to probe kinetic traces at different pump fluences. As the pump energy increased, the kinetic traces of the signals (626 nm) are independent of the pump fluence, which in turn reflects that the relaxation time is not sensitive to pump power, confirming the molecule-like characteristics. Therefore, in contrast with the large-sized NCs Ag_141_ and Au_144_ with SPR [63–65], **Ag102** exhibits discrete absorption bands and molecule-like electron dynamics. Similar to the reported case of Ag_146_ [62], the geometry of **Ag102** resembles a flying saucer, and the anisotropic shape affects the emergence of SPR, delaying the onset of metallic behavior. Besides, the simplified Jablonski diagram provides basic insights into the preferable mechanism of energy release subsequent to the excitation of **Ag102** [66,67], and it shows that non-fluorescent emitters are complemented by energy decay in the form of heat de-excitation, enabling **Ag102** to be employed as excellent photothermal conversion reagents (Fig. 7f).

## DISCUSSION

In summary, we have successfully synthesized a giant 102-nuclei silver NC with a silvery fullerene kernel by a DMF-thermal reaction. The silvery fullerene kernel of Ag_32_ was trapped by a robust anionic layer of (KPO_4_)_10_, which plays an important role in passivating the inner Ag_32_ kernel and supporting the outer Ag_70_ shell. Combined with the *^t^*BuPhS^−^ and CF_3_COO^−^ ligands for regioselective distribution on the surface of the **Ag102**, the whole cluster possesses a *D*_5_*_d_* symmetry. **Ag102** exhibits superior photothermal conversion performance in both solution and crystalline states, and the photothermal conversion efficiency of **Ag102** solution at 450 nm is remarkably high up to 67.1% ± 0.9%. Furthermore, fs-TA studies have substantiated the molecule-like characteristics of **Ag102**, and its photothermal conversion is attributed to non-radiative transitions. Our study provides a cornerstone for further incorporating M into coinage metal NCs, and the fullerene topologic structure of Ag_32_ trapped in silver NC will open up new opportunities for the synthesis of more silvery fullerenes.

## Supplementary Material

nwae192_Supplemental_Files

## Data Availability

Experimental details, computational details, detailed crystallographic structure and data including the CIF file, PXRD and IR are available within the article and its Supplementary information files. Other relevant data are available from the corresponding author upon request. The X-ray crystallographic coordinates for structures reported in this article have been deposited at the Cambridge Crystallographic Data Centre, under deposition number CCDC 2323089 for **Ag102**. These data can be obtained free of charge from the Cambridge Crystallographic Data Centre via www.ccdc.cam.ac.uk/data_request/cif.
