# Supplementary material for: Silvery fullerene in Ag102 nanosaucer
Source: Natl Sci Rev. 2024 Jun 6;11(7):nwae192. doi: 10.1093/nsr/nwae192 (PMC11282957; doi:10.1093/nsr/nwae192)
Supplement: nwae192_Supplemental_Files [file nwae192_supplemental_files.zip › Ag102-SI-V44-R1.docx]

**Supplementary Information (SI)**

**Silvery Fullerene in Ag_102_ Nanosaucer**

Zhi Wang,^1,3^ Yuchen Wang,^2,3^ Chengkai Zhang,^1^ Yan-Jie Zhu,^1^ Ke-Peng Song,^1^ Christine M. Aikens,^*,2^ Chen-Ho Tung,^1^ and Di Sun^*,1^

^1^School of Chemistry and Chemical Engineering, State Key Laboratory of Crystal Materials, Shandong University, Ji’nan 250100, People’s Republic of China.

^2^Department of Chemistry, Kansas State University, Manhattan, Kansas 66506, USA.

^3^Z. W. and Y. W. contributed equally to this work.

**Experiment details.**

Solvents and reagents (Adamas-Beta®) were purchased from Shanghai Titan Scientific Co.,Ltd. Unless otherwise noted, all of the chemicals were reagent grade and used without any further purification. The precursor of {(HNEt_3_)_2_[Ag_10_(*^t^*BuPhS)_12_]}*_n_*^1^ and the ligand of *p*-tert-butylthiacalix[4]arene (H_4_TC4A)^2^ were prepared by following the reported procedure. The high-angle annular dark-field scanning transmission electron microscopy (HAADF-STEM) experiments were carried out at 300 kV using Thermofisher Spectra 300 scanning transmission electron microscope with a probe Cs-corrector. The sample was prepared by the evaporation of picoliter-scaled droplets of **Ag102** in CH_2_Cl_2_ on hydrophobic substrates (carbon-coated copper grid). The PXRD data was acquired by using a Rigaku Oxford Diffraction XtaLAB Synergy diffractometer with Cu radiation (λ = 1.54184 Å). The Powder function of the *CrysAlis^Pro^* software suite^3^ was used to process the PXRD patterns. The elemental analyses (C, H, N contents) were determined on a Vario EL III analyzer. The infrared spectrum was recorded on a Bruker Tensor II spectrophotometer (Bruker Optics GmbH, Ettlingen, Germany) utilizing a single attenuated total reflectance (ATR) accessory covering a wavenumber range from 500 to 4000 cm^-1^. **Ag102** crystals were pressed onto the diamond window by a plane steel cone. The final spectrum was the average of 32 scans accumulated using Bruker’s Opus software 8.1, taken at 4 cm^-1^ resolution. Samples for transient absorption spectroscopy measurements were prepared by dissolving **Ag102** in CHCl_3_ and transferred to a 2-mm path length quartz cuvette (1mg mL^-1^). An Astrella Ti: Sapphire laser system from Coherent was used as a light source, which operates at a 1-kHz repetition rate, generating 70-fs pulses at 800 nm. The ~70-fs pump laser pulse was generated by a regenerative amplifier system and the optical parametric amplifier (Coherent, Solo). A small portion of the laser fundamental was focused into a sapphire plate to produce a supercontinuum in the visible range, which overlapped with the pump in time and space. Multiwavelength transient spectra were recorded at different pump probe delay times (Helios Fire, Ultrafast Systems). Time zero, solvent response, and chirp corrections were used using software supplied by Ultrafast Systems. UV-Vis spectra were recorded on a Thermo Scientific Evolution 220 UV-visible spectrophotometer. Mass spectra (MS) were recorded on a Bruker impact II high definition mass spectrometer, quadrupole and time-of-flight (Q/TOF) modules. The data analysis of mass spectrum was performed based on the isotope distribution patterns using Compass Data Analysis software (Version 4.4). Photothermal measurements were conducted using laser (CNI Laser MDL-F-450-1 W DF61479, MDL-MD-660-1.3 W CE50050 and MDL-N-808-8 W CD40781). The photothermal behavior of the sample was monitored by a thermal imaging camera (FLIR E54). Infrared photos and real-time temperatures were extracted from the video by FLIR tools software. A CHCl_3_ solution of **Ag102** was prepared by dissolving the crystals (2.4 mg) in 1 mL of CHCl_3_. The **Ag102**/match remote ignition material was performed by dispersing **Ag102** crystals (2 mg) in 0.3 mL of ethanol, sonicating for about 10 min, and then spreading evenly on the match surface.

**X-ray Crystallography**

The single crystal of **Ag102** with appropriate dimensions was chosen under an optical microscope and quickly coated with high vacuum grease (Dow Corning Corporation) to prevent decomposition. Single-crystal X-ray diffraction data of **Ag102** was collected on a Rigaku Oxford Diffraction XtaLAB Synergy diffractometer equipped with a HyPix-6000HE area detector at 100 K using Cu Kα (λ = 1.54184 Å) from Photon Jet micro-focus X-ray Source. The diffraction images were processed and scaled using the *CrysAlis^Pro^* software suite.^3^ The structure was solved using the charge-flipping algorithm, as implemented in the program *SUPERFLIP*^4^ and refined by full-matrix least-squares techniques against *F*_o_^2^ using the SHELXL program^5^ through the OLEX2 interface.^6^ Hydrogen atoms at carbon were placed in calculated positions and refined isotropically by using a riding model. Appropriate restraints or constraints were applied to the geometry and the atomic displacement parameters of the atoms in the cluster. The structure was examined using the Addsym subroutine of PLATON^7^ to ensure that no additional symmetry could be applied to the model. Pertinent crystallographic data collection and refinement parameters are collated in Table S2. Selected bond lengths and angles are collated in Table S3.

**Computational methods**

The optical spectrum of the **Ag102** nanocluster (NC) system was calculated at the BP86/TZP (triple-ζ with polarization functions) level of theory^8,9^ using the TDDFT+TB method^10^ in the Amsterdam Modeling Suite (AMS) 2021.102 software.^11^ The solvent effects were taken into consideration using the Conductor like Screening Model (COSMO)^12-14^ and the calculations were performed in water and DMF solvents, respectively. The structure model is based on the crystal structure coordinates and is shown in Figure S15. All *^t^*BuPhS^-^ ligands were substituted by S-H ligands (with a fixed S-H bond length of 1.37 Å) to simplify the model. Ten CF_3_COO^-^ ligands were also removed from the experimental crystal structure, so a +10 charge was assigned to the system. The overall formula of the model compound is [Ag_102_(KPO_4_)_10_(SH)_60_(DMF)_2_]^10+^, which yields an electron count of 12. The calculated optical spectrum shows good agreement with the experimental spectrum. In addition, the calculated spectrum also shows several peaks around 800 nm and 950 nm, and those two excitations are dominated by transitions from orbitals arising primarily from the sulfur atomic orbitals to other orbitals dominated by the sulfur atomic orbitals.

**Synthesis Details**

Method A: {(HNEt_3_)_2_[Ag_10_(*^t^*BuPhS)_12_]}*_n_* (0.05 mmol, 13.7 mg), H_4_TC4A (0.015 mmol, 10.8 mg), and KH_2_PO_4_ (0.058 mmol, 8 mg) were mixed in 1 mL DMF. Following 3 h stirring (800 rpm) at room temperature (20 ^o^C), a solution of CF_3_COOAg (0.1 mmol, 22.1 mg) in DMF (0.5 mL) was added into the above yellow turbid solution. After stirring for 3 h, the mixture was heated slowly to 65 ^o^C and maintained at this temperature for 33 h. Black rod crystals of **Ag102** were formed at the bottom of the bottle after cooling to room temperature with a yield of 10 % (based on {(HNEt_3_)_2_[Ag_10_(*^t^*BuPhS)_12_]}*_n_*). Elemental analyses calc. (found) for **Ag102** (C_626_H_794_Ag_102_F_30_K_10_N_2_O_62_P_10_S_60_): C, 31.89 (31.95); H, 3.45 (3.40); N 0.13 (0.12) %. Selected IR peaks (cm^-1^; Figure S23): 2956 (m), 1643 (m); 1481 (m); 1394 (w); 1357 (w); 1266 (w); 1194 (m); 1114 (m); 1042 (m); 1005 (s); 816 (s); 722 (m); 541 (s).

Method B: Under otherwise identical conditions, replacing KH_2_PO_4_ with K_2_HPO_4_∙3H_2_O (0.035 mmol, 8 mg) also obtained **Ag102** with a yield of 10 % (based on {(HNEt_3_)_2_[Ag_10_(*^t^*BuPhS)_12_]}*_n_*).

**Figure S1:** **HAADF-STEM images of Ag102. Insets: Size distributions of Ag102.**


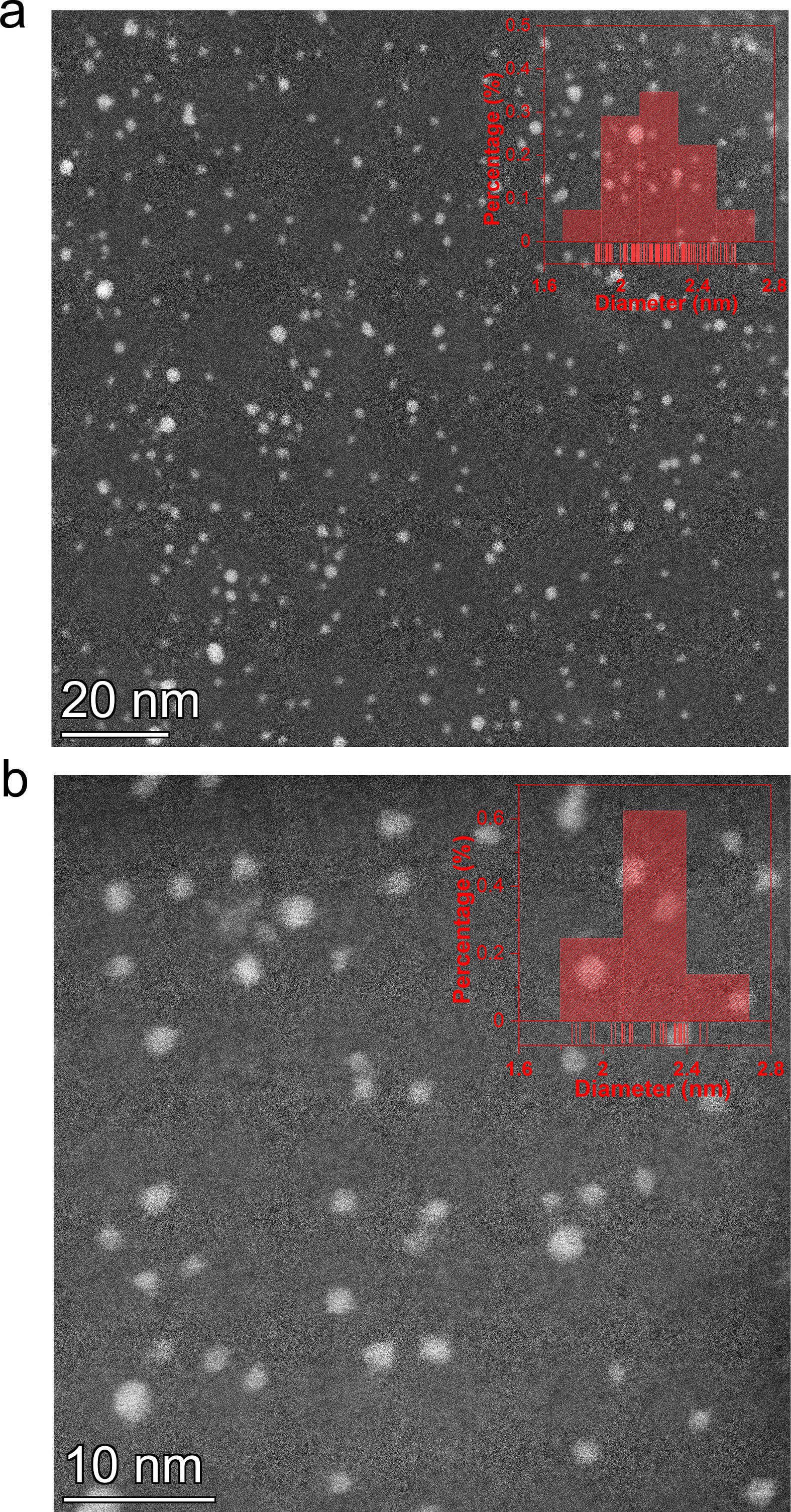


**Figure S2: The molecular packing of Ag102 in the unit cell viewed along *a*, *b* and *c* axis.**


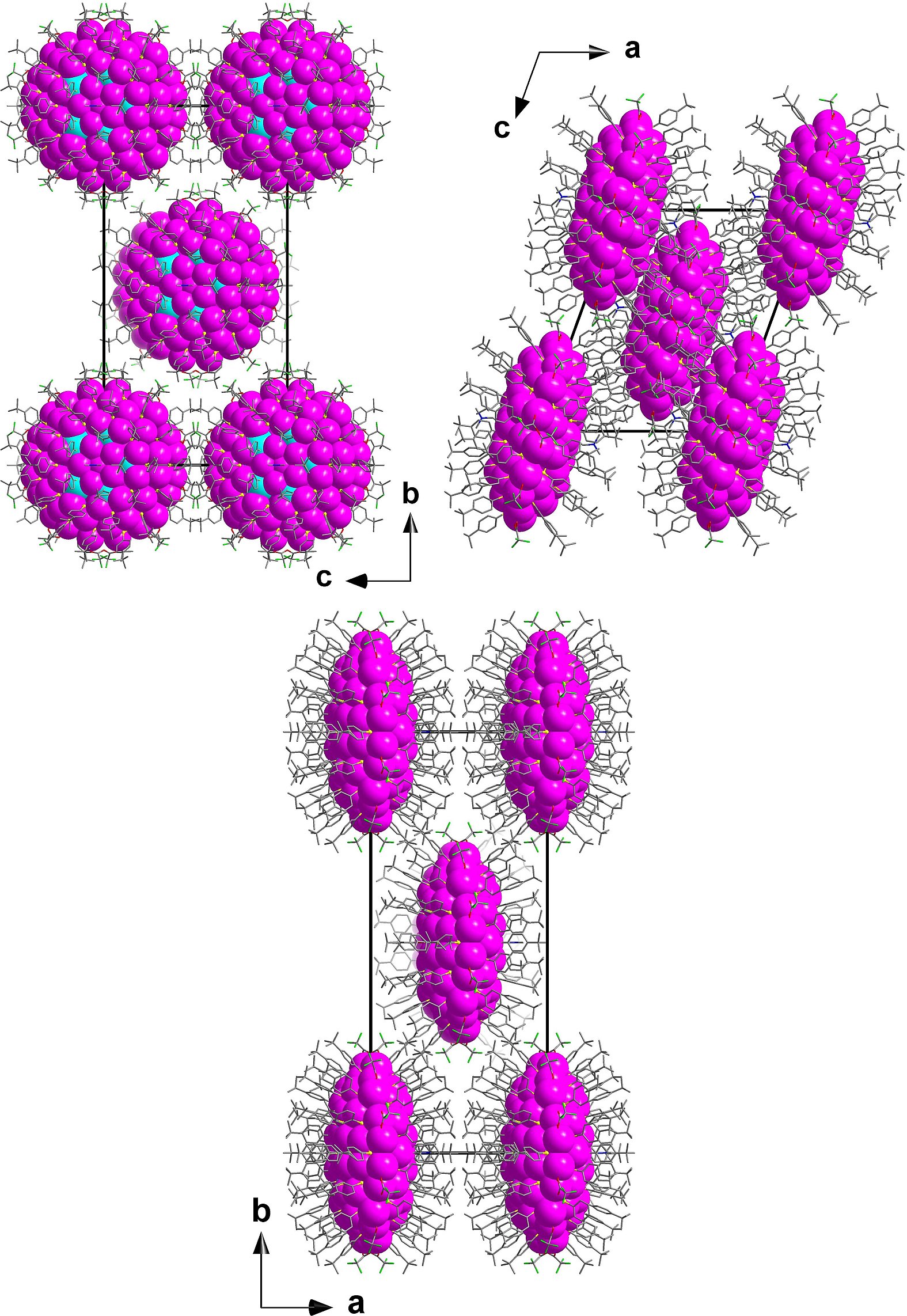


**Figure S3: The top (a) and side (b) views of Ag_12_@Ag_20_, the Ag···Ag distances between Ag_12_ and Ag_20_ represented as two-colored lines, and the Ag···Ag distances in Ag_12_ and Ag_20_ represented as gray lines.**


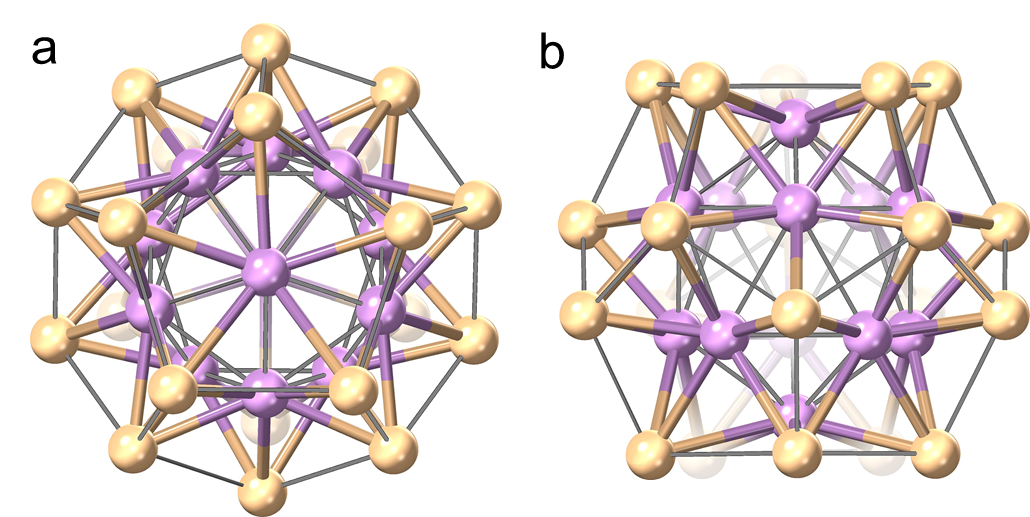


**Figure S4: The size of the Ag_20_ (the distance between two opposite pentagonal faces) (a) C_60_ (b), and [K@Au_12_Sb_20_]^5-^ (c).^15-17^**


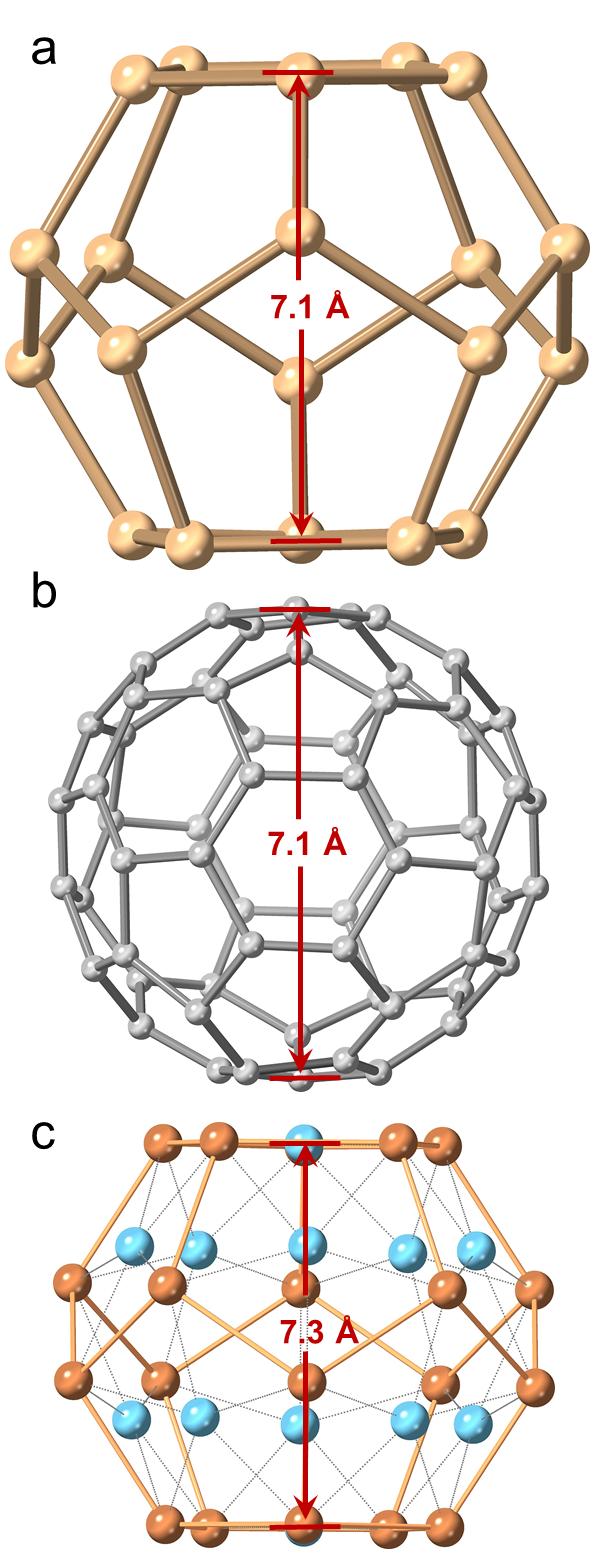


**Figure S5: The Ag_12_@Ag_20_ kernel and 10 K^+^. Color label: lilac, golden, Ag; cyan, K.**


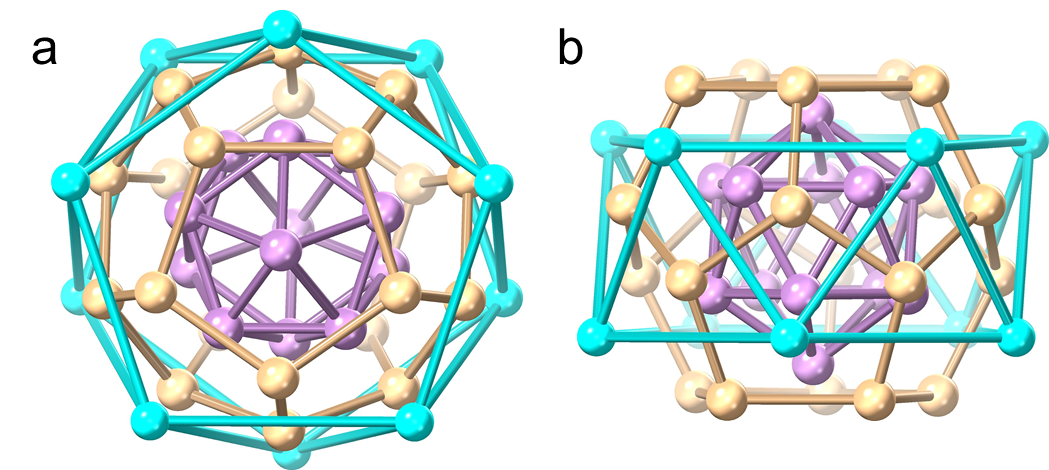


**Figure S6: The K_10_ pentagonal antiprism.** **Color label: cyan, K.**


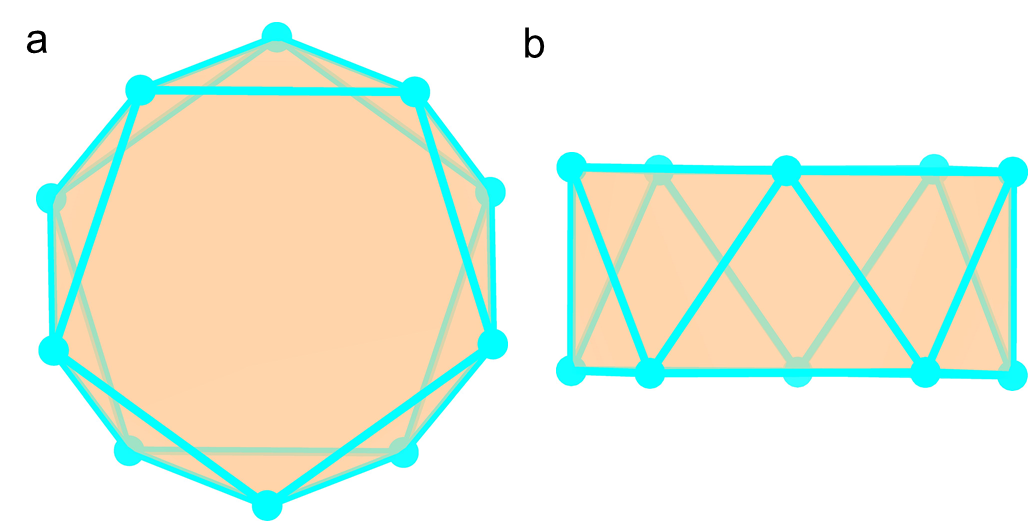


**Figure S7: The coordination patterns of *^t^*BuPhS^-^ ligands with Ag atoms. Color label: purple, Ag; gray, C; yellow, S.**


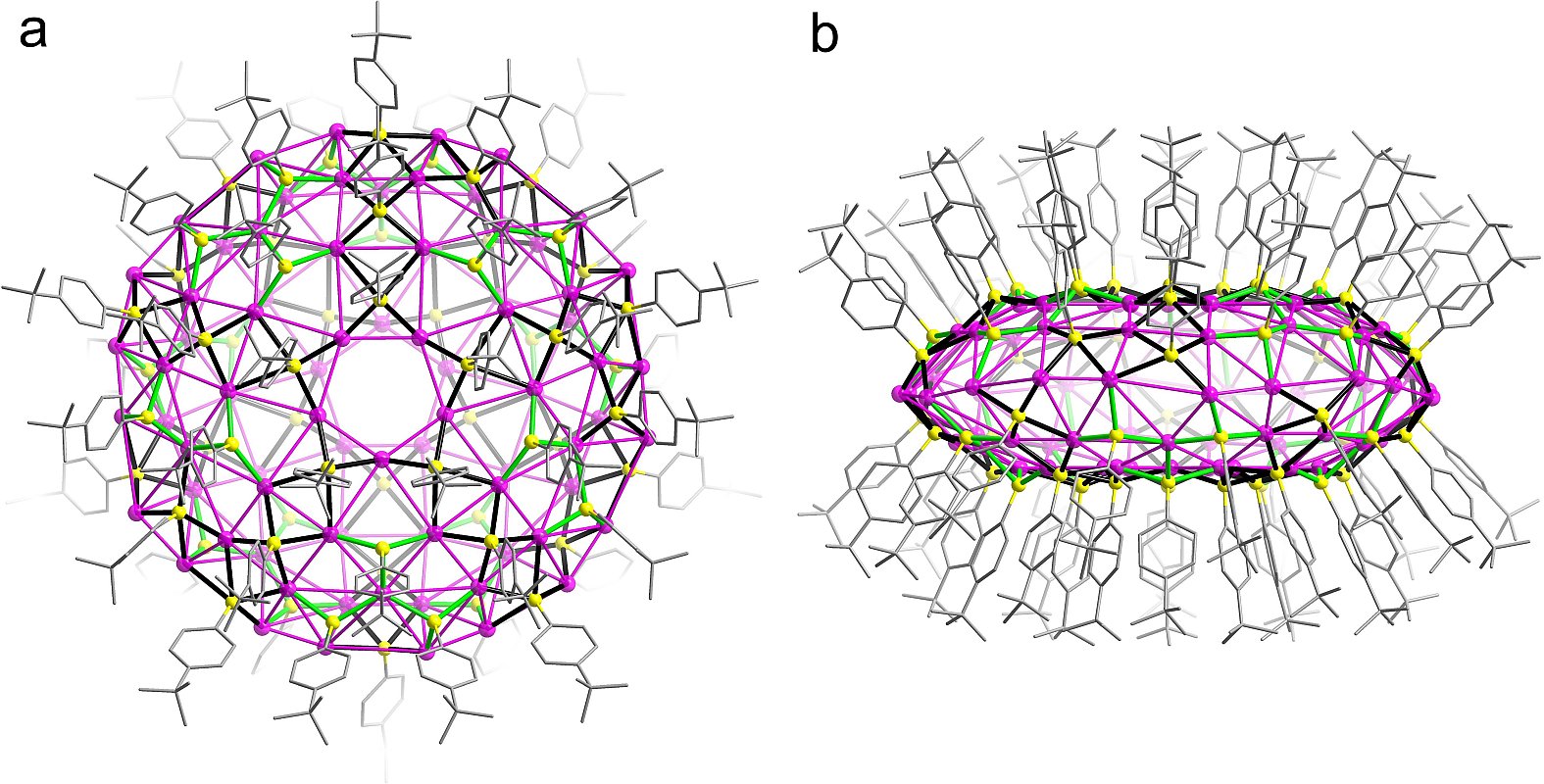


**Figure S8: The coordination patterns of CF_3_COO^-^ ligands and DMF molecules with Ag atoms. Color label: purple, Ag;** **cyan, K; green, F; gray, C; red, O; blue, N; orange tetrahedra, PO_4_^3-^.**


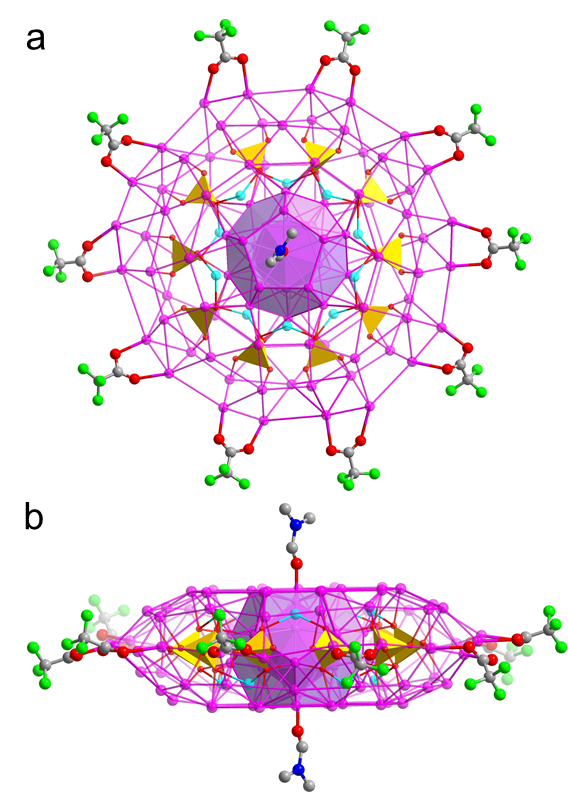


**Figure S9: The coordination pattern of 10 PO_4_^3-^ anions with Ag and K atoms. Color label: purple, green, lilac, Ag; cyan, K; red, O; orange tetrahedra, PO_4_^3-^.**


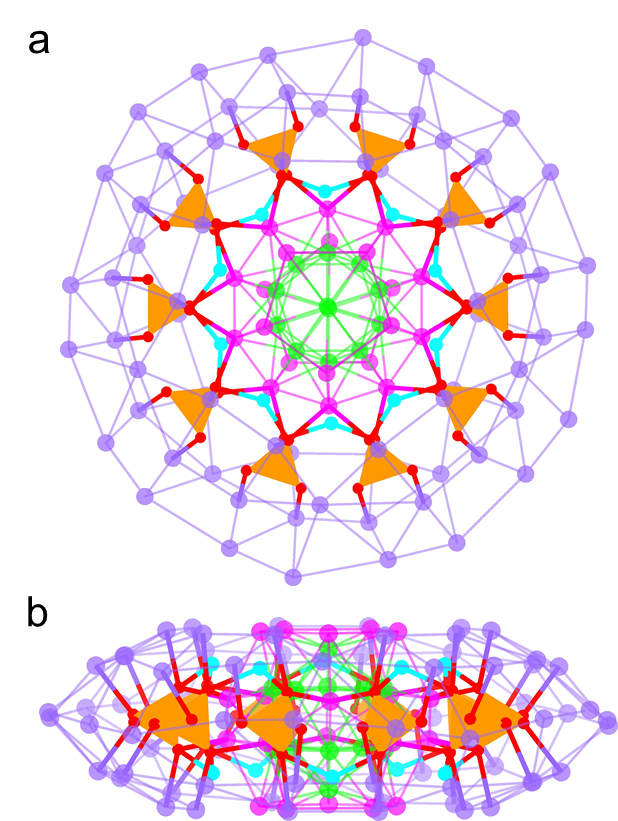


**Figure S10: Torsion angle of two Ag_10_ (a) and Ag_15_ (b) rings. Color label: purple, blue, green, Ag.**


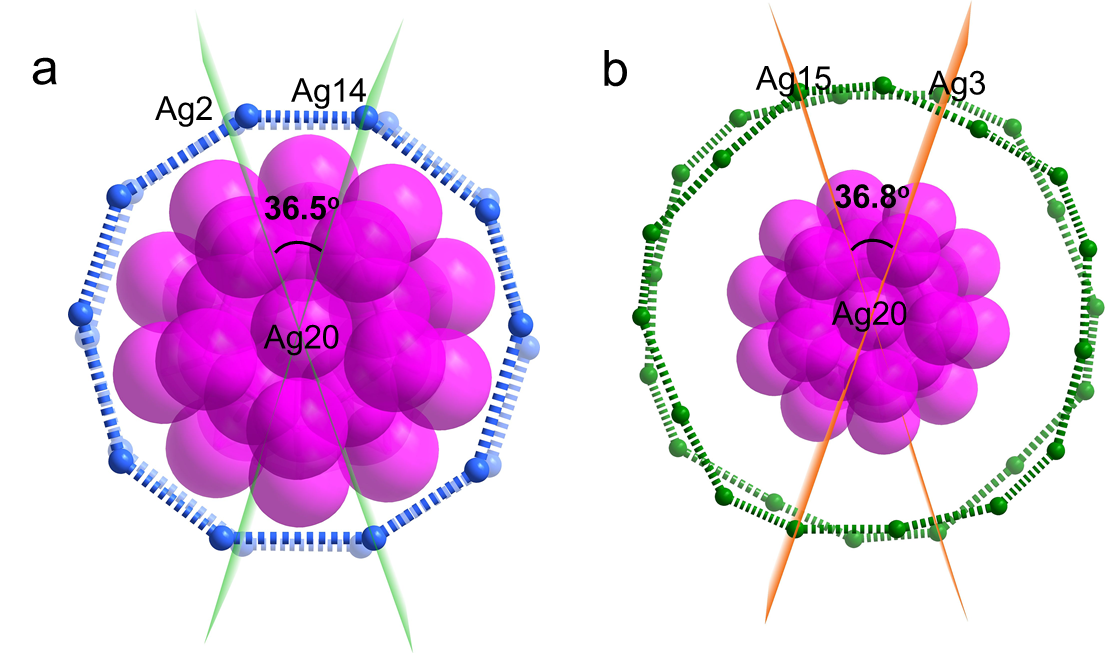


**Figure S11: The ESI-MS of Ag102 dissolved in CH_2_Cl_2_-CH_3_OH mixed solvents in positive ion mode.**


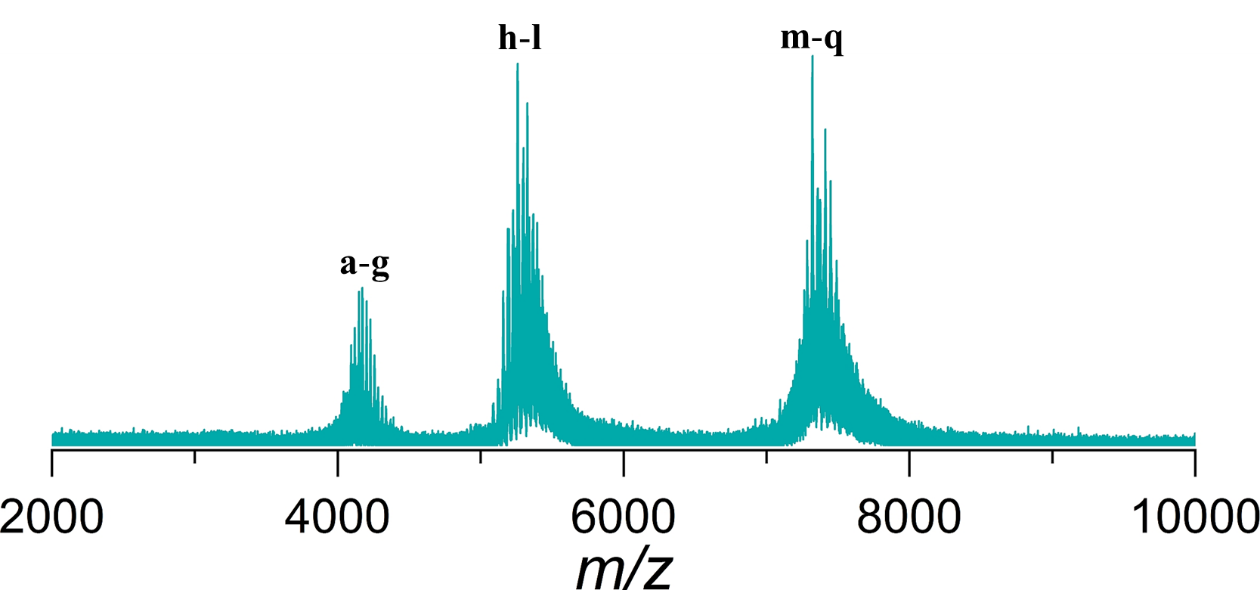


**Figure S12: The experimental (green trace) and simulated (orange trace) isotope patterns of m-q species (Δ=Ag_12_@Ag_20_@(KPO_4_)_10_).**
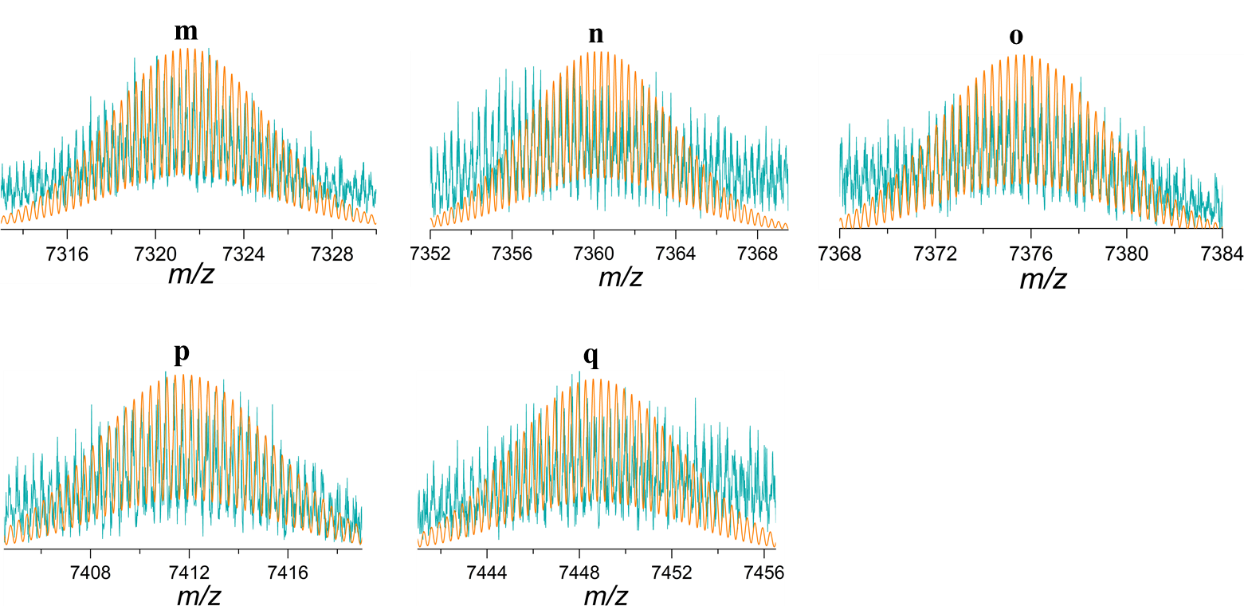


| **Species** | **Assignment** | **Exp. *m/z*** | **Sim. *m/z*** |
| --- | --- | --- | --- |
| **m** | {**Δ**@Ag_65_(*^t^*BuPhS)_60_(CF_3_COO)_2_(H_2_O)}^3+^ | 7321.3829 | 7321.4556 |
| **n** | {**Δ**@Ag_66_(*^t^*BuPhS)_58_(CF_3_COO)_5_(H_2_O)}^3+^ | 7360.3853 | 7360.3597 |
| **o** | {**Δ**@Ag_66_(*^t^*BuPhS)_58_(CF_3_COO)_5_(CH_3_OH)_2_}^3+^ | 7375.7479 | 7375.7070 |
| **p** | {**Δ**@Ag_66_(*^t^*BuPhS)_60_(CF_3_COO)_3_(CH_3_OH)(H_2_O)_2_}^3+^ | 7411.7061 | 7411.7645 |
| **q** | {**Δ**@Ag_66_(*^t^*BuPhS)_60_(CF_3_COO)_3_(DMF)_2_(CH_3_OH)}^3+^ | 7448.3520 | 7448.4594 |

**Figure S13: The experimental (green trace) and simulated (orange trace) isotope patterns of a-g species** **(Δ=Ag_12_@Ag_20_****@****(KPO_4_)_10_).**


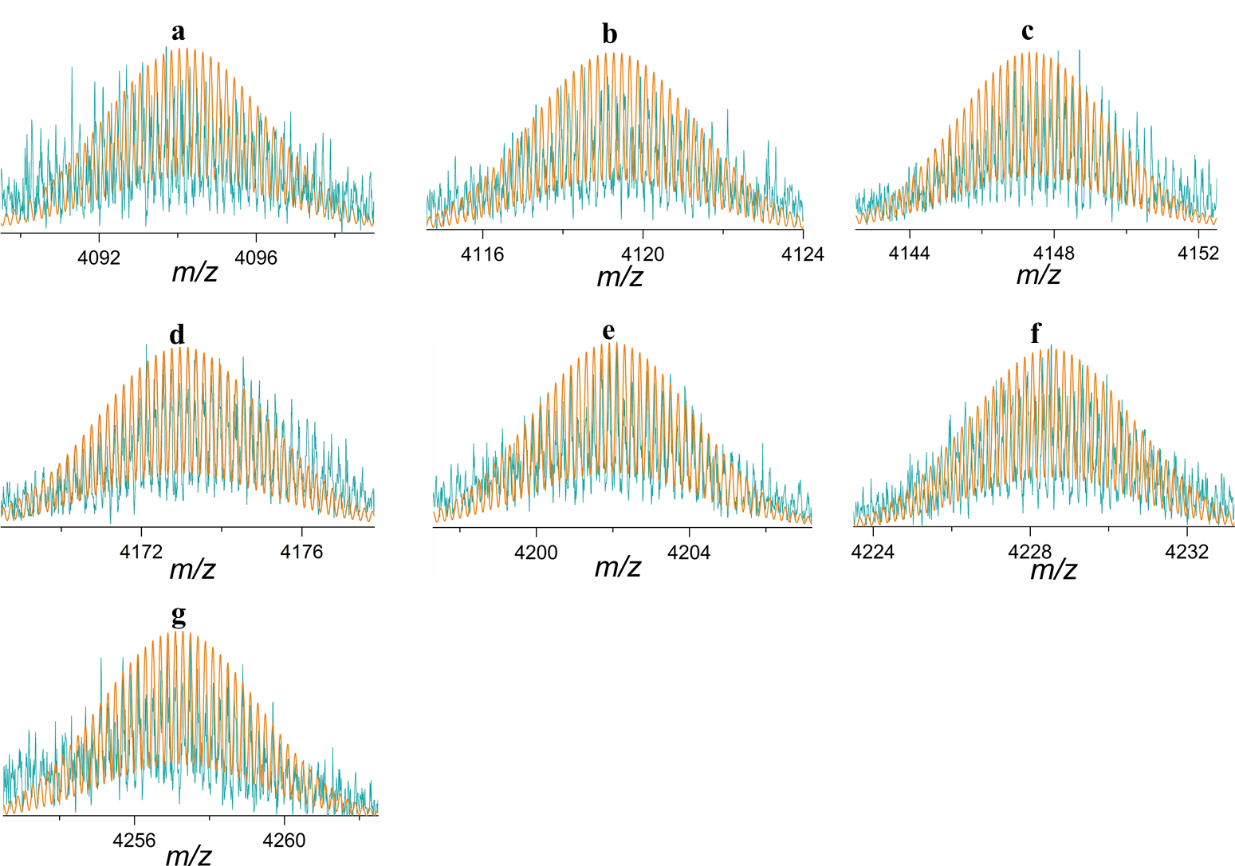


| **Species** | **Assignment** | **Exp. *m/z*** | **Sim. *m/z*** |
| --- | --- | --- | --- |
| **a** | {**Δ**@Ag_61_(*^t^*BuPhS)_53_(CF_3_COO)_3_}^5+^ | 4094.3110 | 4094.2405 |
| **b** | {**Δ**@Ag_61_(*^t^*BuPhS)_54_(CF_3_COO)_2_(DMF)}^5+^ | 4119.3512 | 4119.2689 |
| **c** | {**Δ**@Ag_61_(*^t^*BuPhS)_56_(DMF)(H_2_O)_2_}^5+^ | 4147.2975 | 4147.3086 |
| **d** | {**Δ**@Ag_63_(*^t^*BuPhS)_50_(CF_3_COO)_8_(DMF)(H_2_O)_2_}^5+^ | 4172.9482 | 4172.9580 |
| **e** | {**Δ**@Ag_62_(*^t^*BuPhS)_57_(DMF)(H_2_O)_2_}^5+^ | 4202.1103 | 4202.1044 |
| **f** | {**Δ**@Ag_63_(*^t^*BuPhS)_56_(CF_3_COO)_2_(DMF)}^5+^ | 4228.5259 | 4228.4604 |
| **g** | {**Δ**@Ag_63_(*^t^*BuPhS)_57_(CF_3_COO)(DMF)_2_(H_2_O)}^5+^ | 4257.2899 | 4257.2909 |

**Figure S14:** **The experimental (green trace) and simulated (orange trace) isotope patterns of h-l species (Δ=Ag_12_@Ag_20_@(KPO_4_)_10_).**


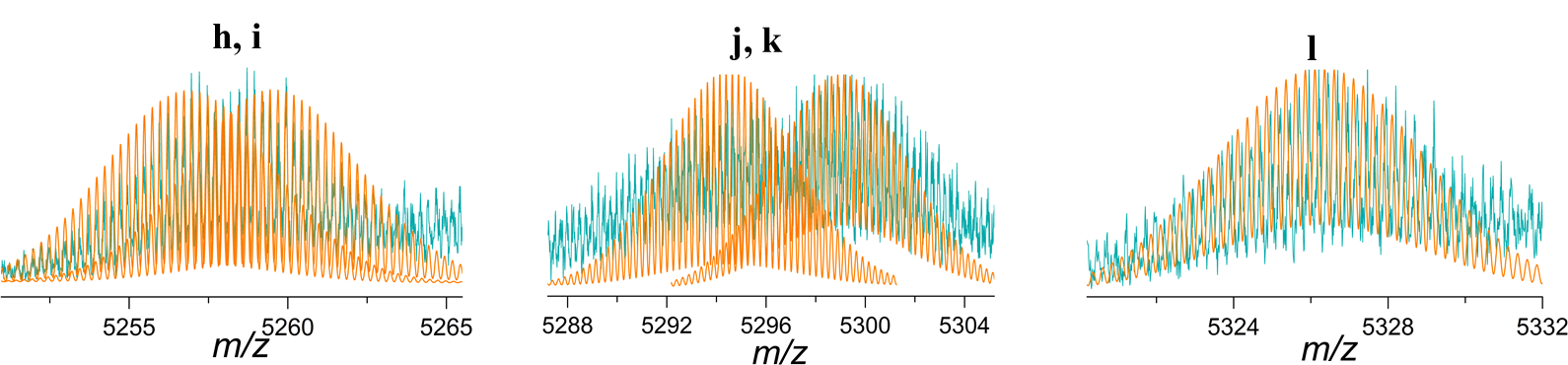


| **Species** | **Assignment** | **Exp. *m/z*** | **Sim. *m/z*** |
| --- | --- | --- | --- |
| **h** | {**Δ**@Ag_63_(*^t^*BuPhS)_52_(CF_3_COO)_7_(H_2_O)_3_}^4+^ | 5256.9616 | 5256.9778 |
| **i** | {**Δ**@Ag_63_(*^t^*BuPhS)_51_(CF_3_COO)_8_(DCM)(CH_3_OH)}^4+^ | 5259.4582 | 5259.4422 |
| **j** | {**Δ**@Ag_62_(*^t^*BuPhS)_57_(CF_3_COO)(DMF)_2_(H_2_O)}^4+^ | 5294.6586 | 5294.6375 |
| **k** | {**Δ**@Ag_62_(*^t^*BuPhS)_57_(CF_3_COO)(DMF)_2_(H_2_O)_2_}^4+^ | 5299.2153 | 5299.1401 |
| **l** | {**Δ**@Ag_63_(*^t^*BuPhS)_58_(CF_3_COO)(H_2_O)}^4+^ | 5326.4461 | 5326.3558 |

**Figure S15: Structure of the [Ag_102_(KPO_4_)_10_(SH)_60_(DMF)_2_]^10+^ system. Color label: purple, Ag; cyan, K; orange, P; blue, N; yellow, S; gray, C; red, O; white, H.**


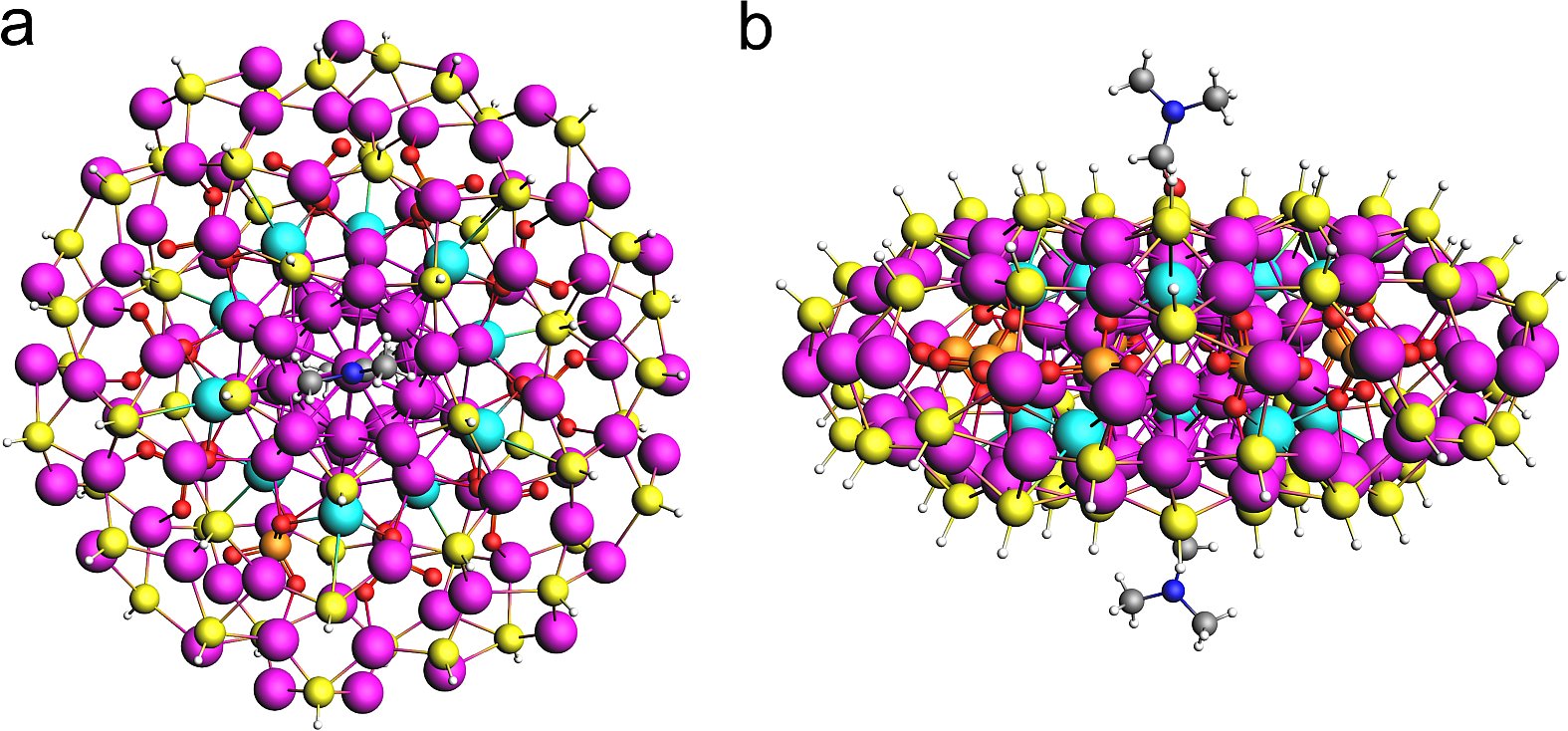


**Figure S16:** **The comparable UV-Vis absorption spectra of the CHCl_3_ solution of Ag102 before (red line) and after (blue line) photothermal conversion** **at a concentration of 6.25 µM.**


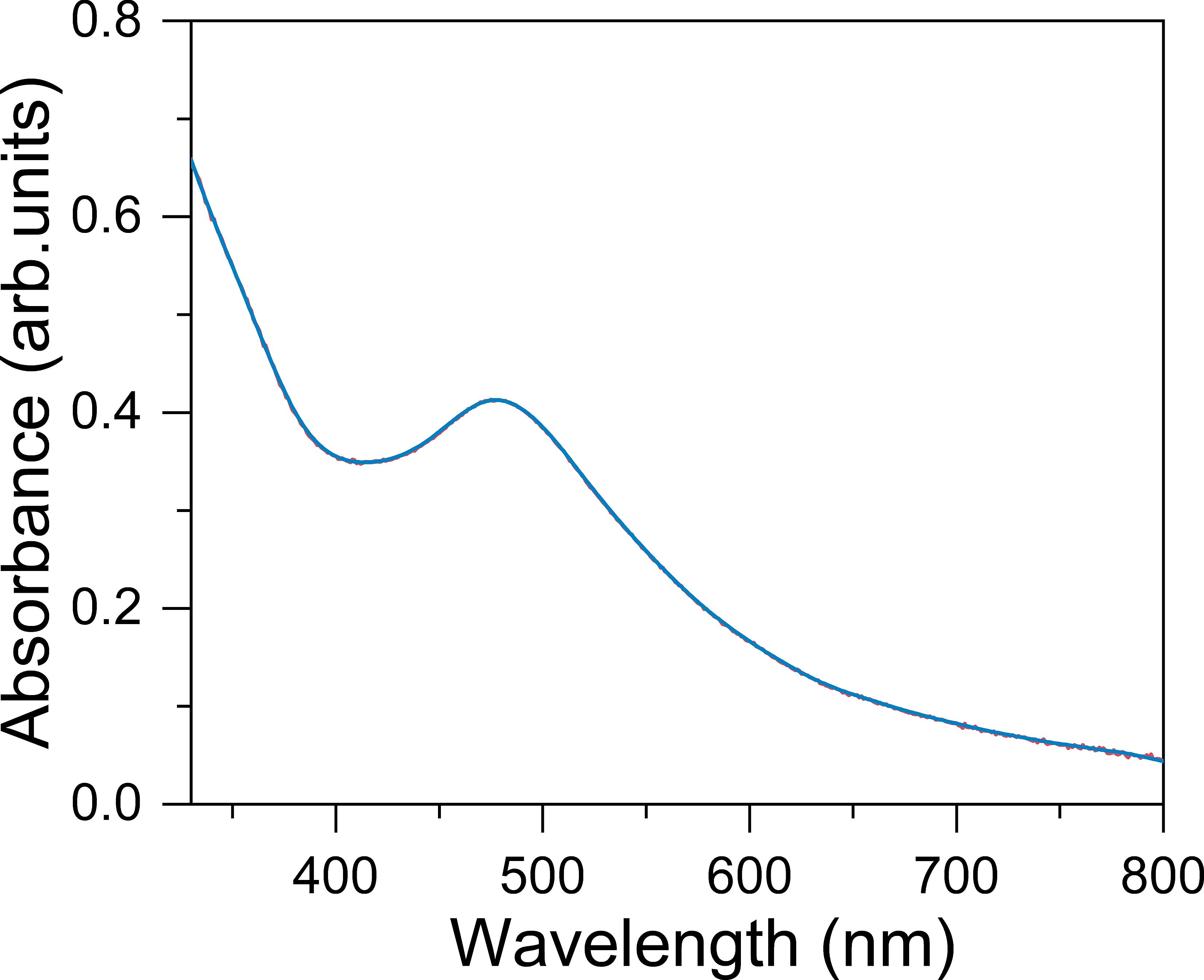


**Figure S17: (a, c, and e) Heating and cooling curve of the CHCl_3_ solution of Ag102 at a concentration of 100 µM** **under 450 nm laser irradiation; (b, d, and f) Linear fit of ln*θ*-*T*. Three experiments on the photothermal conversion conducted in parallel.**


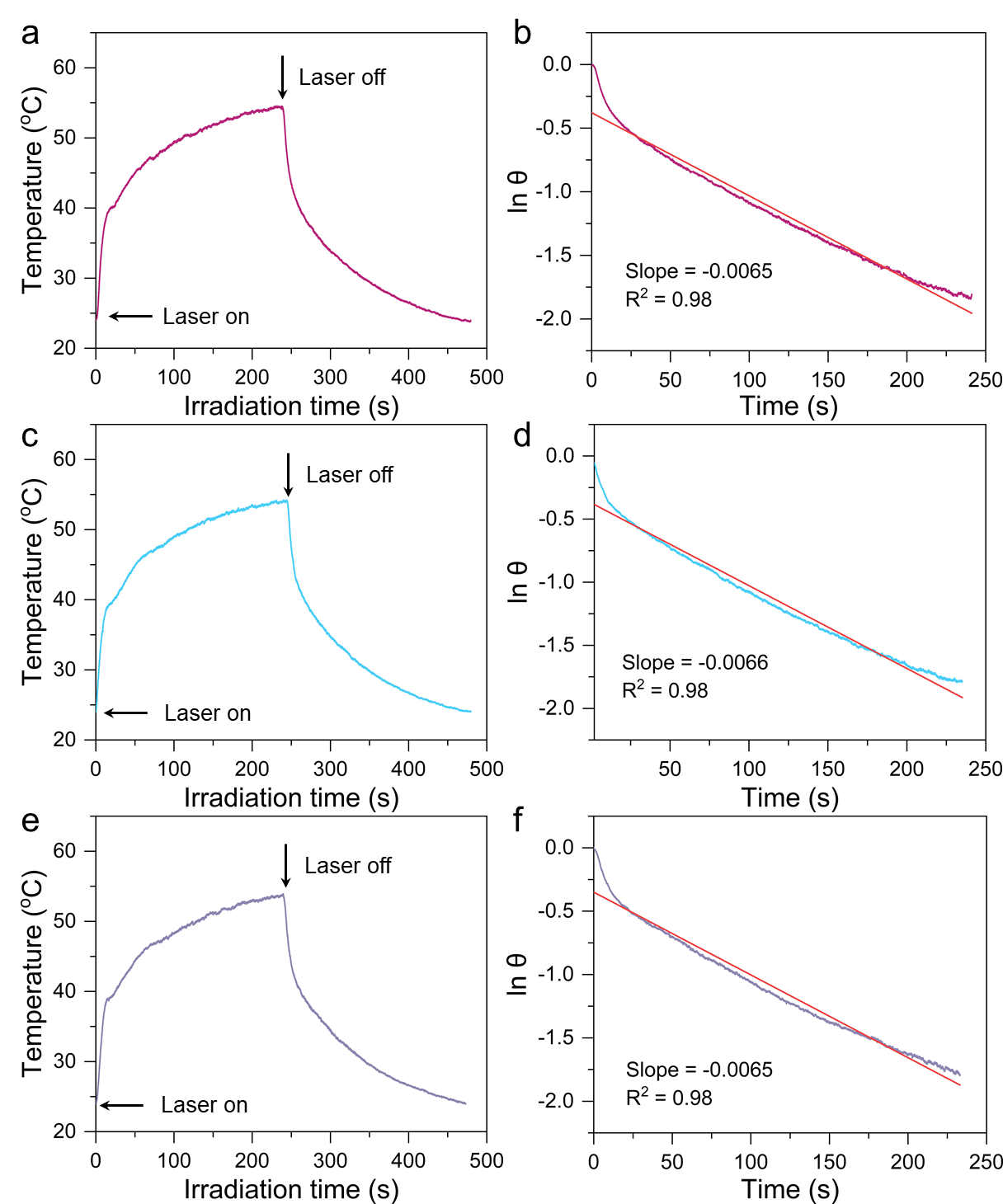


The photothermal conversion efficiency (*η*) can be calculated by the following equations.^18,19^

$$\eta=\frac{{hs(\Delta T}_{sample-}{\Delta T}_{solvent})}{I(1-{10}^{-A})}$$

$$hs=\frac{\sum mC_{P}}{{}_{s}}$$

$${}_{s}=\frac{-t}{ln}$$

$$=\frac{(T_{amb}-T)}{(T_{amb}-T_{max})}$$

where *h* is the heat transfer coefficient, *s* is the surface area of the container, *A* is absorbance of **Ag102** at different wavelength, *I* is the laser power, *m* is the mass of sample, *C_p_* is the specific heat capacity of the solvent and *t* is the time of the cooling process. The *η* of the CHCl_3_ solution of **Ag102** at a concentration of 100 µM under 450 nm laser irradiation was calculated.

For parallel experimental 1 (Figure S17a and S17b): A linear fit of *lnθ*-*T* yielded a slope of -0.0065, by which *τ_s_* was calculated as 153.8 s (slope = -1/*τ_s_*). *ΣmC_p_* = *ρ*(CHCl_3_)·*V*(CHCl_3_)·*C_p_*(CHCl_3_) = 0.5 mL × 1.48 g/mL × 1.189 J/(g ^o^C^-1^) = 0.88 J ^o^C^-1^. Therefore, *hs* = 0.88 / 153.8 = 5.7 × 10^-3^ J·^o^C^-1^·s^-1^. Δ*T_sample_* = 36.4 ^o^C. Δ*T_solvent_* = 1 ^o^C. *A*_1_ = 0.38 × 16 = 6.08 (Figure S16). Eventually, *η*_1_ = 5.7 × 10^-3^ × (36.4 - 1) / [0.3 × (1 - 10^-6.08^)] = 67.3 %.

For parallel experimental 2 (Figure S17c and S17d): A linear fit of *lnθ*-*T* yielded a slope of -0.0066, by which *τ_s_* was calculated as 151.5 s (slope = -1/*τ_s_*). *ΣmC_p_* = *ρ*(CHCl_3_)·*V*(CHCl_3_)·*C_p_*(CHCl_3_) = 0.5 mL × 1.48 g/mL × 1.189 J/(g ^o^C^-1^) = 0.88 J ^o^C^-1^. Therefore, *hs* = 0.88 / 151.5 = 5.8 × 10^-3^ J·^o^C^-1^·s^-1^. Δ*T_sample_* = 36.1 ^o^C. Δ*T_solvent_* = 1 ^o^C. *A*_1_ = 0.38 × 16 = 6.08 (Figure S16). Eventually, *η*_1'_ = 5.8 × 10^-3^ × (36.1 - 1) / [0.3 × (1 - 10^-6.08^)] = 67.9 %.

For parallel experimental 3 (Figure S17e and S17f): A linear fit of *lnθ*-*T* yielded a slope of -0.0065, by which *τ_s_* was calculated as 153.8 s (slope = -1/*τ_s_*). *ΣmC_p_* = *ρ*(CHCl_3_)·*V*(CHCl_3_)·*C_p_*(CHCl_3_) = 0.5 mL × 1.48 g/mL × 1.189 J/(g ^o^C^-1^) = 0.88 J ^o^C^-1^. Therefore, *hs* = 0.88 / 153.8 = 5.7 × 10^-3^ J·^o^C^-1^·s^-1^. Δ*T_sample_* = 35.8 ^o^C. Δ*T_solvent_* = 1 ^o^C. *A*_1_ = 0.38 × 16 = 6.08 (Figure S16). Eventually, *η*_1"_ = 5.7 × 10^-3^ × (35.8 - 1) / [0.3 × (1 - 10^-6.08^)] = 66.1 %.

Based on the above-mentioned results, the photothermal conversion efficiency of **Ag102** at 450 nm is 67.1±0.9% (The error is based on the standard deviation of three parallel experiments).

**Figure S18: (a, c, and e) Heating and cooling curve of the CHCl_3_ solution of Ag102 at a concentration of 100 µM** **under 660 nm laser irradiation; (b, d, and f) Linear fit of ln*θ*-*T*. Three experiments on the photothermal conversion conducted in parallel.**


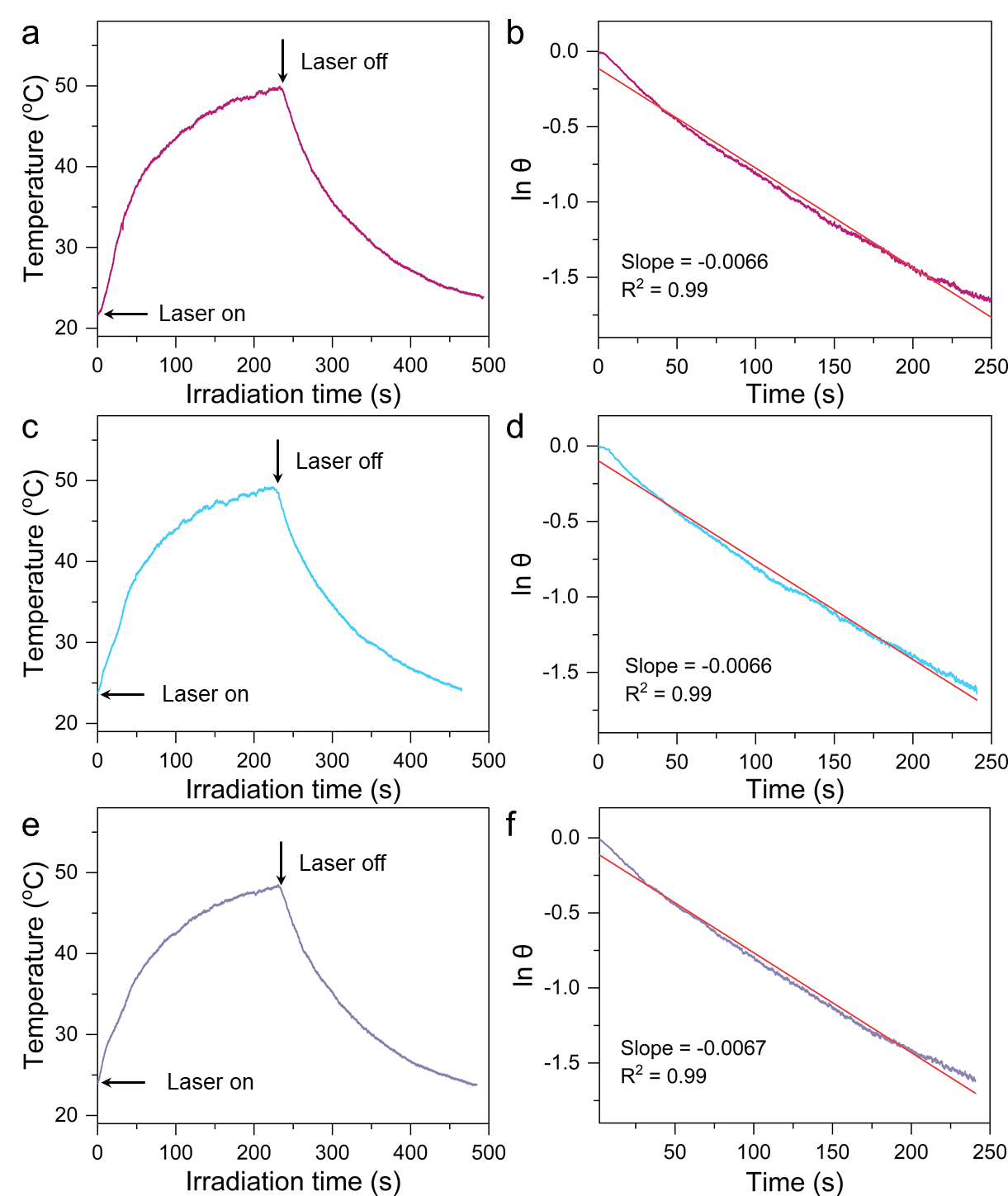


The *η* of the CHCl_3_ solution of **Ag102** at a concentration of 100 µM under 660 nm laser irradiation was calculated.

For parallel experimental 1 (Figure S18a and S18b): A linear fit of *lnθ*-*T* yielded a slope of -0.0066, by which *τ_s_* was calculated as 151.5 s (slope = -1/*τ_s_*). *ΣmC_p_* = *ρ*(CHCl_3_)·*V*(CHCl_3_)·*C_p_*(CHCl_3_) = 0.5 mL × 1.48 g/mL × 1.189 J/(g ^o^C^-1^) = 0.88 J ^o^C^-1^. Therefore, *hs* = 0.88 / 151.5 = 5.8 × 10^-3^ J·^o^C^-1^·s^-1^. Δ*T_sample_* = 31.9 ^o^C. Δ*T_solvent_* = 1 ^o^C. *A*_2_ = 0.09 × 16 = 1.44 (Figure S16). Eventually, *η*_2_ = 5.8 × 10^-3^ × (31.9 - 1) / [0.3 × (1 - 10^-1.44^)] = 62.0 %.

For parallel experimental 2 (Figure S18c and S18d): A linear fit of *lnθ*-*T* yielded a slope of -0.0066, by which *τ_s_* was calculated as 151.5 s (slope = -1/*τ_s_*). *ΣmC_p_* = *ρ*(CHCl_3_)·*V*(CHCl_3_)·*C_p_*(CHCl_3_) = 0.5 mL × 1.48 g/mL × 1.189 J/(g ^o^C^-1^) = 0.88 J ^o^C^-1^. Therefore, *hs* = 0.88 / 151.5 = 5.8 × 10^-3^ J·^o^C^-1^·s^-1^. Δ*T_sample_* = 31.2 ^o^C. Δ*T_solvent_* = 1 ^o^C. *A*_2_ = 0.09 × 16 = 1.44 (Figure S16). Eventually, *η*_2'_ = 5.8 × 10^-3^ × (31.2 - 1) / [0.3 × (1 - 10^-1.44^)] = 60.6 %.

For parallel experimental 3 (Figure S18e and S18f): A linear fit of *lnθ*-*T* yielded a slope of -0.0067, by which *τ_s_* was calculated as 149.3 s (slope = -1/*τ_s_*). *ΣmC_p_* = *ρ*(CHCl_3_)·*V*(CHCl_3_)·*C_p_*(CHCl_3_) = 0.5 mL × 1.48 g/mL × 1.189 J/(g ^o^C^-1^) = 0.88 J ^o^C^-1^. Therefore, *hs* = 0.88 / 149.3 = 5.9 × 10^-3^ J·^o^C^-1^·s^-1^. Δ*T_sample_* = 30.5 ^o^C. Δ*T_solvent_* = 1 ^o^C. *A*_2_ = 0.09 × 16 = 1.44 (Figure S16). Eventually, *η*_2"_ = 5.9 × 10^-3^ × (30.5 - 1) / [0.3 × (1 - 10^-1.44^)] = 60.2 %.

Based on the above-mentioned results, the photothermal conversion efficiency of **Ag102** at 660 nm is 60.9±0.9% (The error is based on the standard deviation of three parallel experiments).

**Figure S19: (a, c, and e) Heating and cooling curve of the CHCl_3_ solution of Ag102 at a concentration of 100 µM** **under 808 nm laser irradiation; (b, d, and f) Linear fit of ln*θ*-*T*. Three experiments on the photothermal conversion conducted in parallel.**


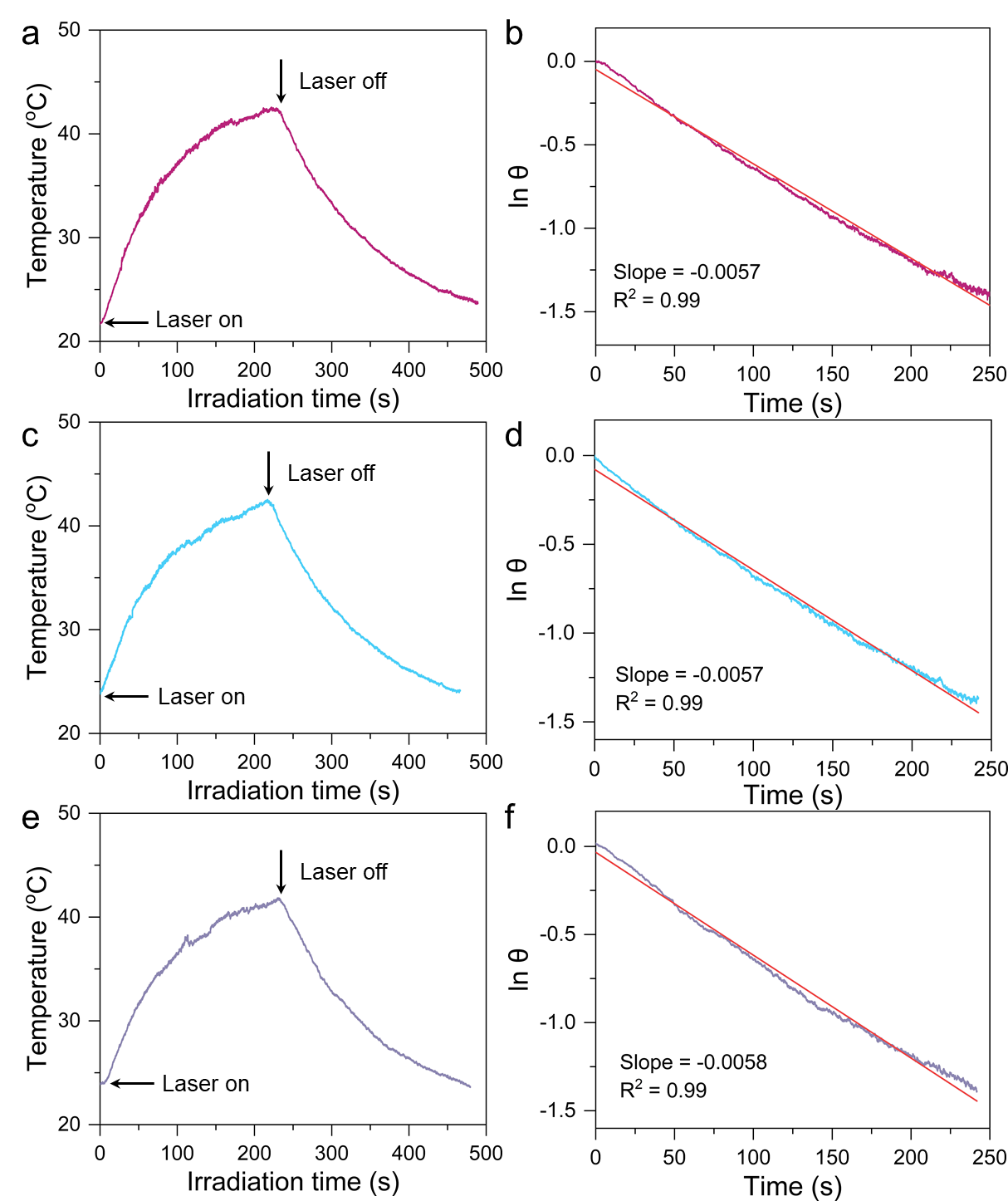


The *η* of the CHCl_3_ solution of **Ag102** at a concentration of 100 µM under 808 nm laser irradiation was calculated.

For parallel experimental 1 (Figure S19a and S19b): A linear fit of *lnθ*-*T* yielded a slope of -0.0057, by which *τ_s_* was calculated as 175.4 s (slope = -1/*τ_s_*). *ΣmC_p_* = *ρ*(CHCl_3_)·*V*(CHCl_3_)·*C_p_*(CHCl_3_) = 0.5 mL × 1.48 g/mL × 1.189 J/(g ^o^C^-1^) = 0.88 J ^o^C^-1^. Therefore, *hs* = 0.88 / 175.4 = 5.0 × 10^-3^ J·^o^C^-1^·s^-1^. Δ*T_sample_* = 24.4 ^o^C. Δ*T_solvent_* = 1 ^o^C. *A*_3_ = 0.04 × 16 = 0.64 (Figure S16). Eventually, *η*_3_ = 5.0 × 10^-3^ × (24.4 - 1) / [0.3 × (1 - 10^-0.64^)] = 50.6 %.

For parallel experimental 2 (Figure S19c and S19d): A linear fit of *lnθ*-*T* yielded a slope of -0.0057, by which *τ_s_* was calculated as 175.4 s (slope = -1/*τ_s_*). *ΣmC_p_* = *ρ*(CHCl_3_)·*V*(CHCl_3_)·*C_p_*(CHCl_3_) = 0.5 mL × 1.48 g/mL × 1.189 J/(g ^o^C^-1^) = 0.88 J ^o^C^-1^. Therefore, *hs* = 0.88 / 175.4 = 5.0 × 10^-3^ J·^o^C^-1^·s^-1^. Δ*T_sample_* = 24.0 ^o^C. Δ*T_solvent_* = 1 ^o^C. *A*_3_ = 0.04 × 16 = 0.64 (Figure S16). Eventually, *η*_3'_ = 5.0 × 10^-3^ × (24.0 - 1) / [0.3 × (1 - 10^-0.64^)] = 49.7 %.

For parallel experimental 3 (Figure S19e and S19f): A linear fit of *lnθ*-*T* yielded a slope of -0.0058, by which *τ_s_* was calculated as 172.4 s (slope = -1/*τ_s_*). *ΣmC_p_* = *ρ*(CHCl_3_)·*V*(CHCl_3_)·*C_p_*(CHCl_3_) = 0.5 mL × 1.48 g/mL × 1.189 J/(g ^o^C^-1^) = 0.88 J ^o^C^-1^. Therefore, *hs* = 0.88 / 172.4 = 5.1 × 10^-3^ J·^o^C^-1^·s^-1^. Δ*T_sample_* = 23.8 ^o^C. Δ*T_solvent_* = 1 ^o^C. *A*_3_ = 0.04 × 16 = 0.64 (Figure S16). Eventually, *η*_3"_ = 5.1 × 10^-3^ × (23.8 - 1) / [0.3 × (1 - 10^-0.64^)] = 50.3 %.

Based on the above-mentioned results, the photothermal conversion efficiency of **Ag102** at 808 nm is 50.2±0.5% (The error is based on the standard deviation of three parallel experiments).

**Figure S20:** **Photothermal conversion efficiency of reported silver NCs, gold NCs, silver nanoparticles (NPs), and alloy materials.^20-28^**


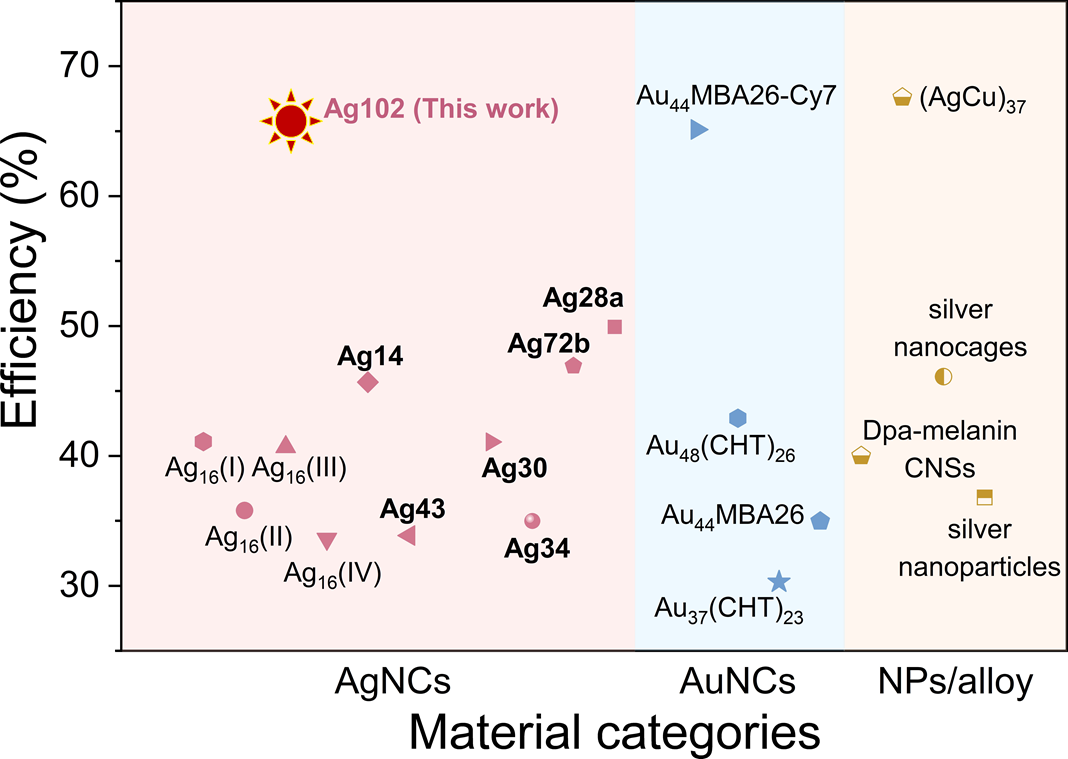


**Figure S21: Diffuse reflectance UV-Vis spectrum of Ag102. Insert: Diffuse reflectance UV-Vis spectrum of Kubelka-Munk function *versus* energy (eV) of Ag102.**


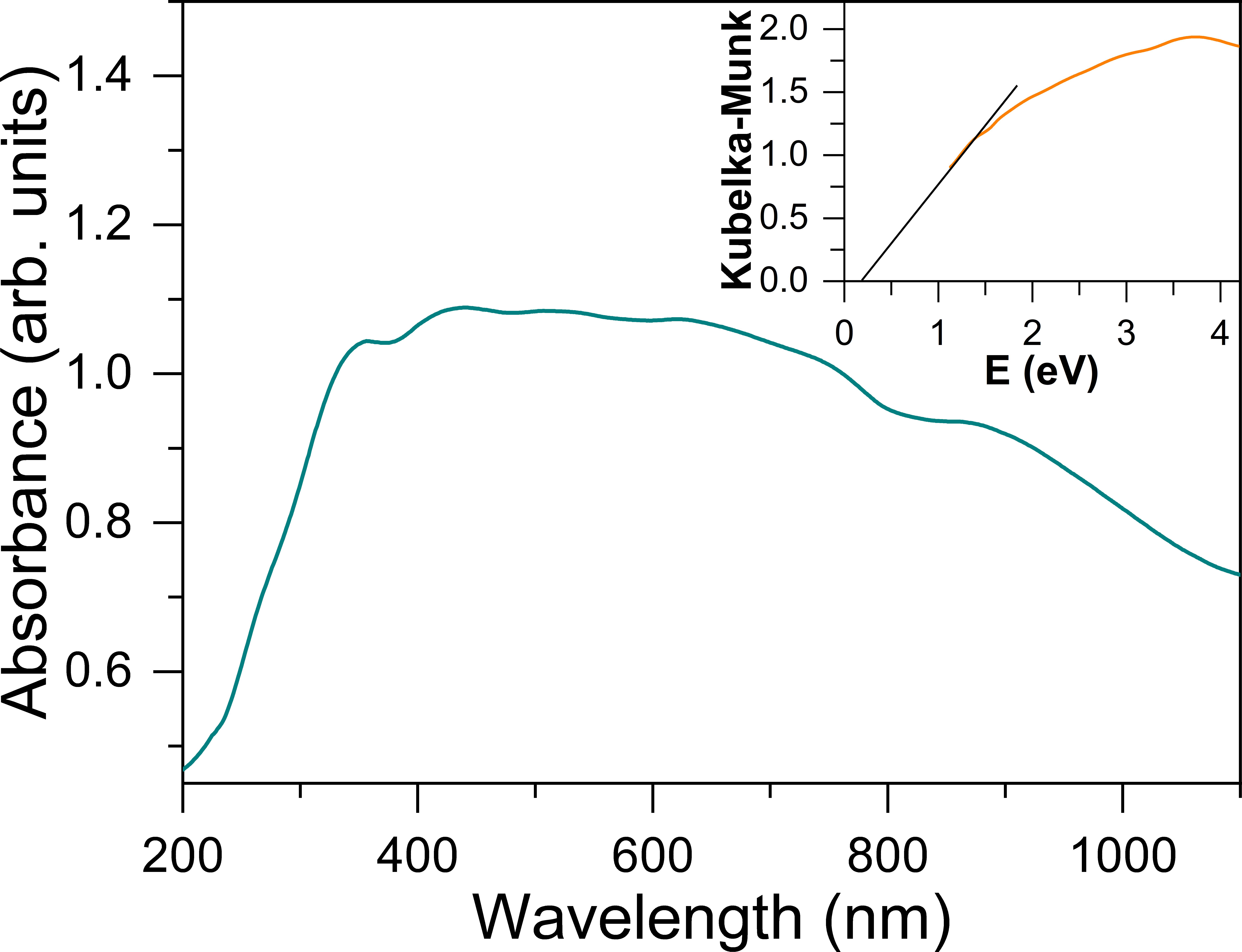


**Figure S22: The plots of temperature evolution *versus* irradiation time for match (a-c) and Ag102/match (d-f) with different laser wavelengths and power at a distance of 20 cm.**


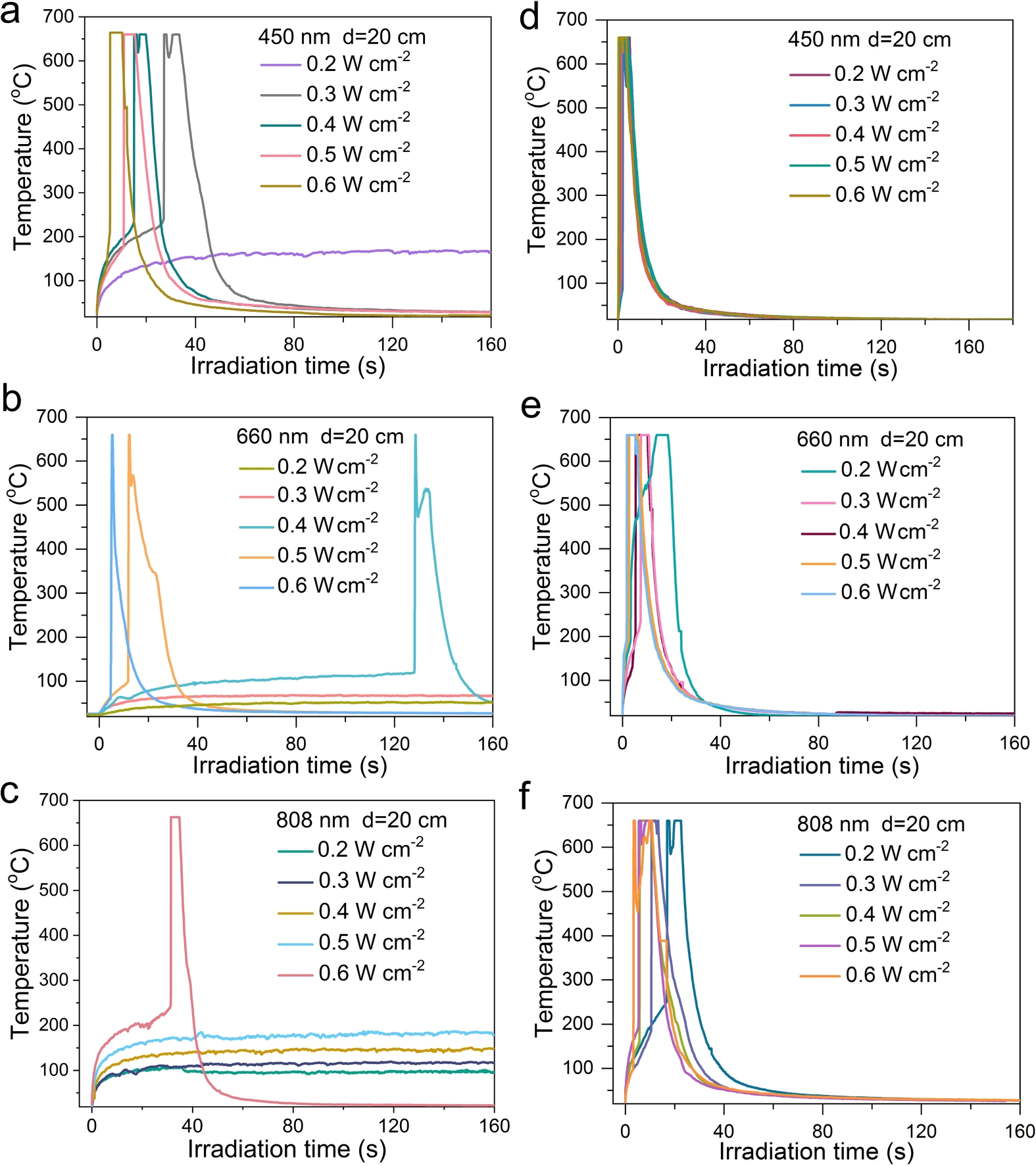


**Figure S23: The IR spectrum of Ag102.**


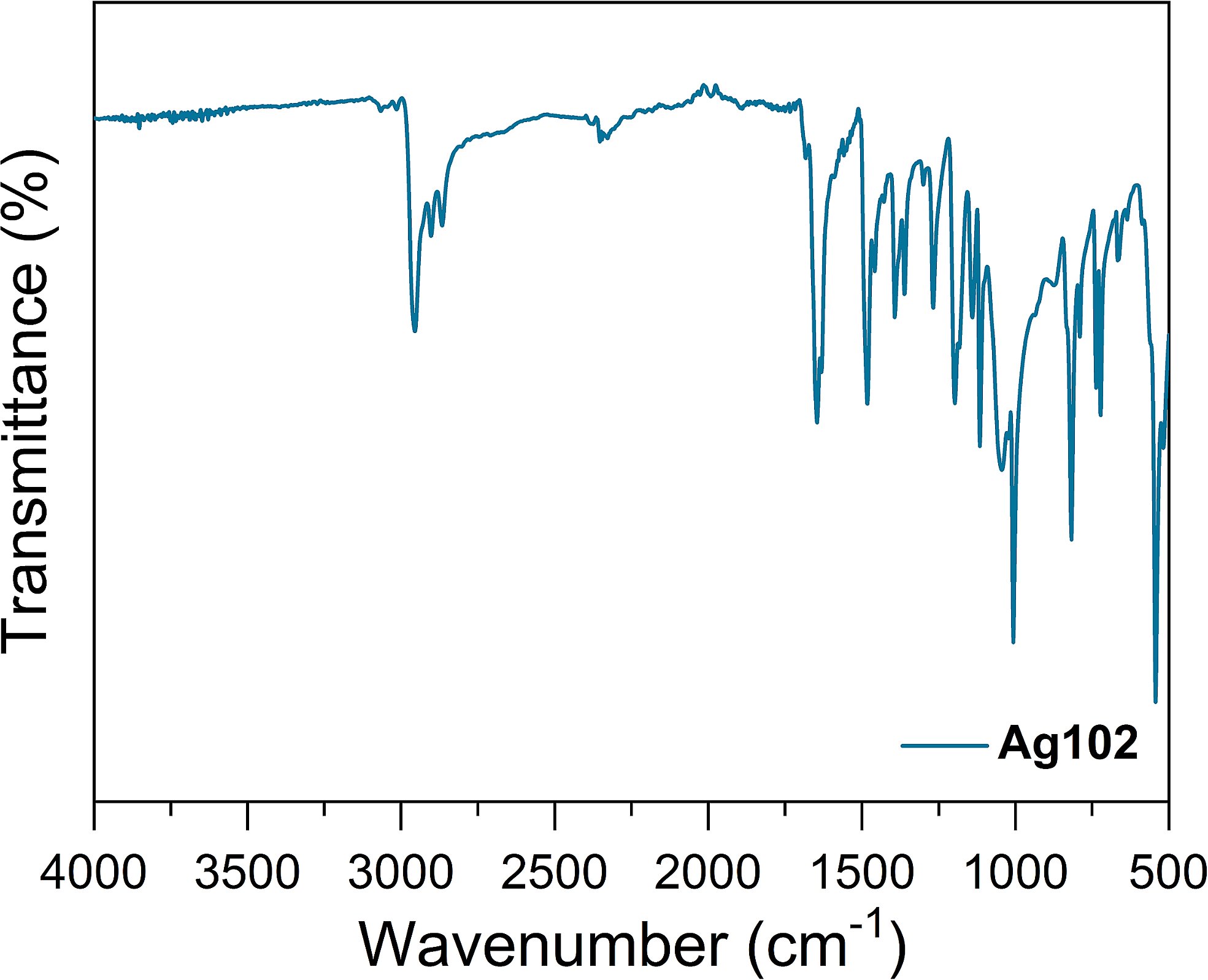


In the IR spectrum of **Ag102**, the band at 1643 cm^-1^ is indicative of the presence of the CF_3_COO^-^ ligand. The infrared bands at 541 and 1005 cm^-1^ are assigned to the PO_4_^3-^ anion.

**Figure S24: The compared PXRD pattern of Ag102.**


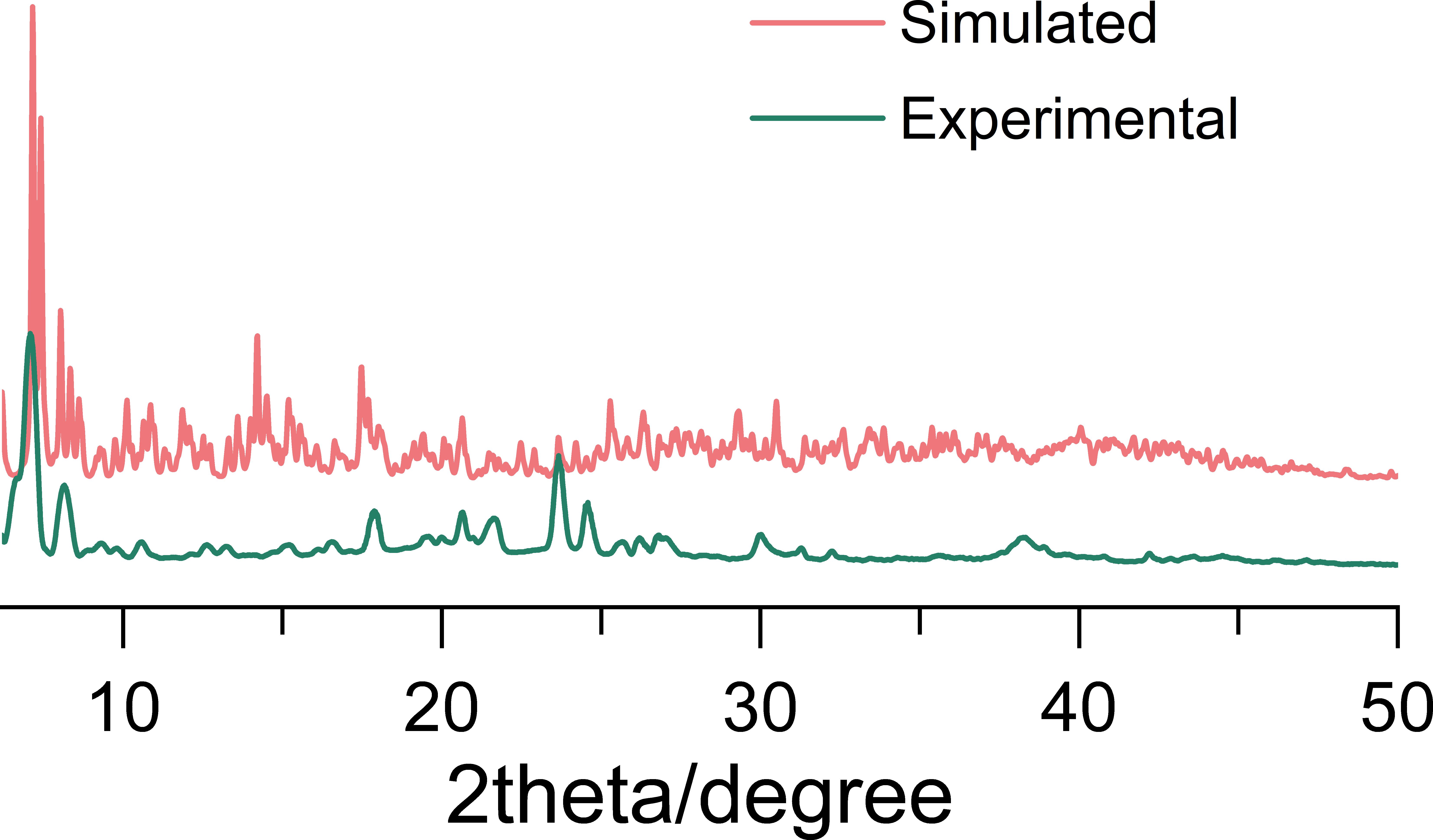


**Table S1: Reported silver NCs with subvalent kernels** **by DMF** **reduction.**

| Compounds | Kernel | Total structure with all ligands omitted | Kernel Structure | References |
| --- | --- | --- | --- | --- |
| [Ag_6_@(MoO_4_)_7_@Ag_56_(MoO_4_)_2_(*^i^*PrS)_28_(*p*-TOS)_14_(DMF)_4_] | Ag_6_ | 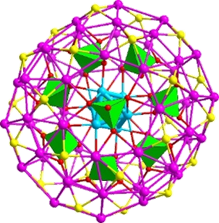 | 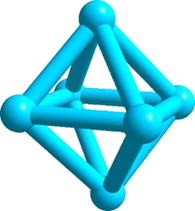 | 29 |
| [Ag_6_@(MoO_4_)_7_@Ag_60_(MoO_4_)_2_(*^i^*PrS)_28_(PhCOO)_18_(CH_3_OH)_2_] | Ag_6_ | 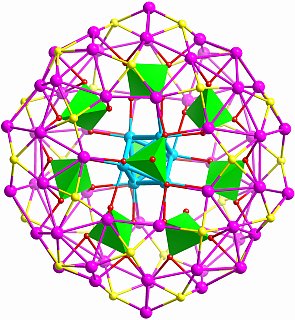 | 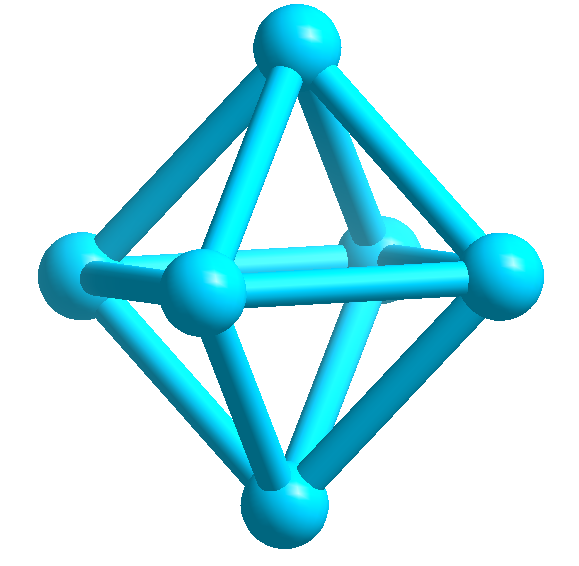 | 30 |
| [Ag_6_@(CrO_4_)_8_@Ag_52_(*^i^*PrS)_30_(DMF)_14_]∙10BF_4_∙2DMF | Ag_6_ | 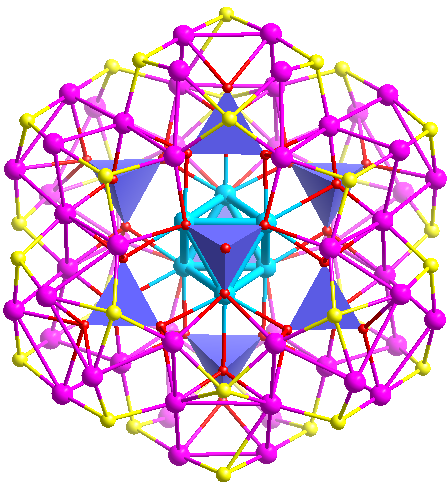 | 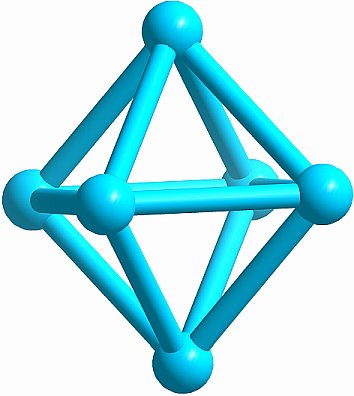 | 31 |
| [(CO_3_)_2_Cl_2_@Ag_7_@Ag_37_(*^i^*PrPhS)_31_(dppm)_6_·3ClO_4_·DMF] | Ag_7_ | 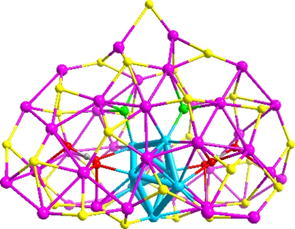 | 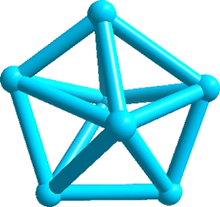 | 32 |
| [Ag_10_@(MoO_4_)_7_@Ag_60_(*^t^*BuC_6_H_4_S)_33_(mbc)_18_(DMF)O_2_]·DMF·H_2_O | Ag_10_ | 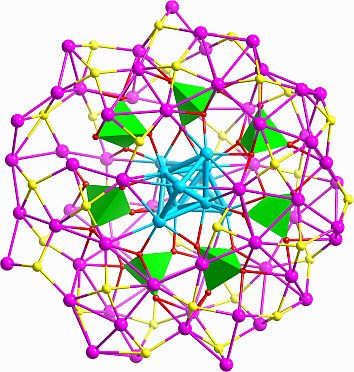 | 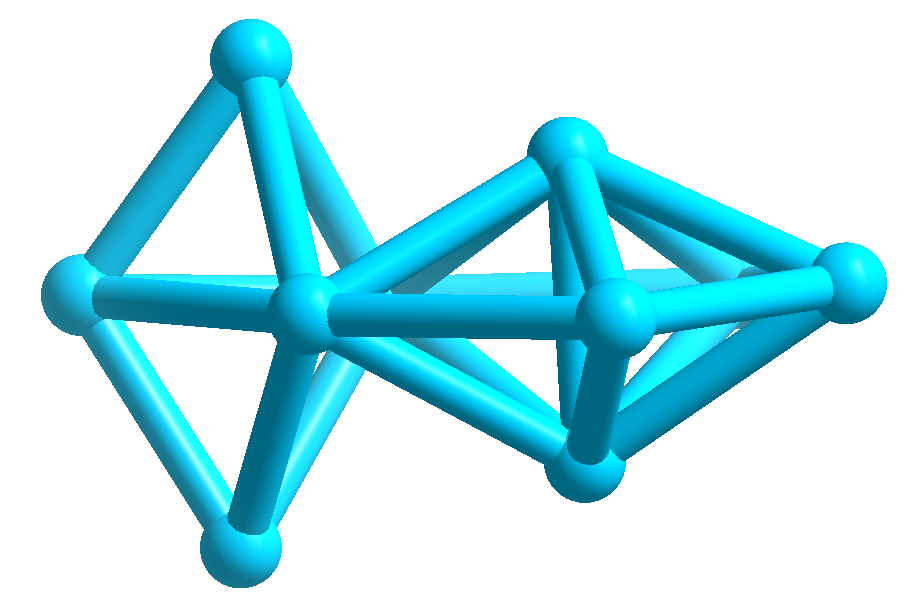 | 33 |
| [Ag_10_@(Mo_7_O_26_)_2_@Ag_70_(MoO_4_)_2_(CyhS)_36_(CF_3_SO_3_)_16_(DMF)_6_]∙2DMF∙4*^n^*PrOH | Ag_10_ | 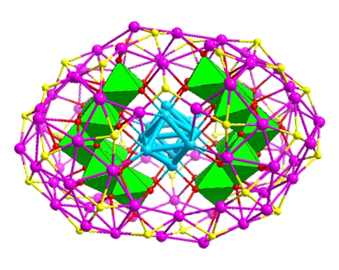 | 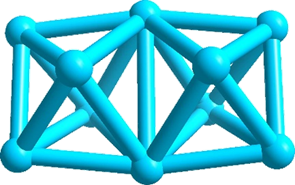 | 34 |
| [Ag_10_@(W_7_O_26_)_2_@Ag_74_S_2_(*^i^*PrS)_40_(*^n^*PrCOO)_18_]∙2CH_3_OH | Ag_10_ | 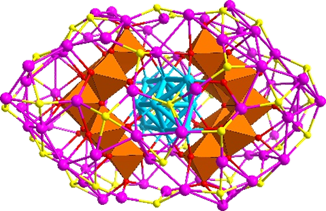 | 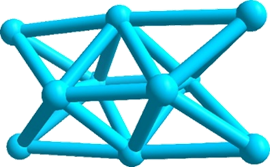 | 35 |
| {[(CO_3_)_2_·H_2_O]_2_@Ag_11_@Ag_43_(*^t^*BuPhS)_36_(dppm)_6_(NHdmpym)_2_(DMF)](ClO_4_)_4_·DMF} | Ag_11_ | 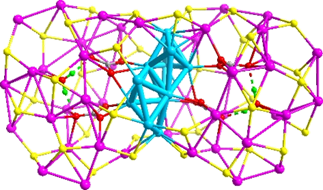 | 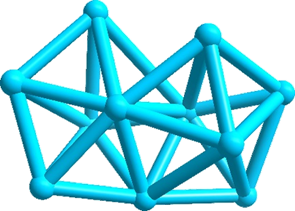 | 32 |
| [Ag_13_@Ag_76_S_16_(CyhS)_42_(*p*-NH_2_-PhAsO_3_)_4_]·3NO_3_ | Ag_13_ | 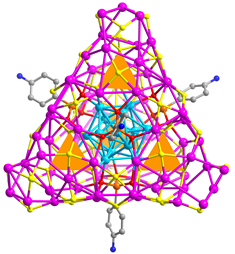 | 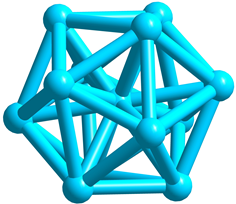 | 36 |
| [Ag_12_@Ag_20_@(KPO_4_)_10_@Ag_70_(*^t^*BuPhS)_60_(CF_3_COO)_10_(DMF)_2_] | Ag_32_ | 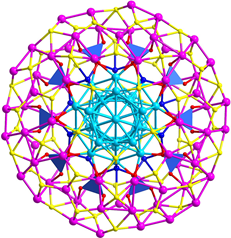 | 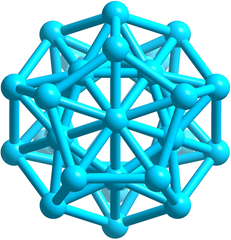 | This work |

**Table S2: Crystal data collection and structure refinement for Ag102.**

| Compound | **Ag102** |
| --- | --- |
| Empirical formula | C_626_H_794_Ag_102_F_30_K_10_N_2_O_62_P_10_S_60_ |
| Formula weight | 23535.62 |
| Temperature/K | 100.00(10) |
| Crystal system | Monoclinic |
| Space group | *I*2/*m* |
| a/Å | 25.3259(5) |
| b/Å | 56.8196(16) |
| c/Å | 30.8259(7) |
| α/° | 90 |
| β/° | 109.913(2) |
| γ/° | 90 |
| Volume/Å^3^ | 41706.5(18) |
| Z | 2 |
| ρ_calc_g/cm^3^ | 1.874 |
| μ/mm^‑1^ | 21.188 |
| F(000) | 22848.0 |
| Radiation | Cu K_α_ (λ = 1.54184 Å) |
| 2Θ range for data collection/° | 5.574 to 140.61 |
| Index ranges | -30 ≤ h ≤ 18, -65 ≤ k ≤ 65, -36 ≤ l ≤ 37 |
| Reflections collected | 120632 |
| Independent reflections | 37393 [R_int_= 0.0694, R_sigma_= 0.0691] |
| Data/parameters | 37393/2697 |
| Goodness-of-fit on F^2^ | 1.072 |
| Final R indexes [I>=2σ (I)] | R_1_= 0.0844, wR_2_= 0.2430 |
| Final R indexes [all data] | R_1_= 0.1143, wR_2_= 0.2724 |

**Table S3: Selected bond distances (Å) and angles (°) for Ag102.**

| Ag1-Ag18 | 3.311(3) | Ag16-S12^2^ | 2.541(5) |
| --- | --- | --- | --- |
| Ag1-O14 | 2.375(7) | Ag17-S3 | 2.558(4) |
| Ag1-S1 | 2.468(3) | Ag17-S11 | 2.537(5) |
| Ag1-S3 | 2.434(4) | Ag17-S15 | 2.539(6) |
| Ag2-Ag3 | 3.087(3) | Ag18-Ag18^2^ | 3.218(3) |
| Ag2-O8 | 2.370(8) | Ag18-O15 | 2.437(9) |
| Ag2-S3 | 2.437(4) | Ag18-S1 | 2.706(4) |
| Ag2-S4 | 2.942(4) | Ag18-S9 | 2.610(4) |
| Ag2-S5 | 2.474(4) | Ag18-S15 | 2.422(5) |
| Ag3-Ag4 | 2.997(3) | Ag19-Ag20 | 2.9599(14) |
| Ag3-O7 | 2.502(11) | Ag19-Ag22^2^ | 3.3592(11) |
| Ag3-S5 | 2.458(4) | Ag19-Ag22 | 3.3592(11) |
| Ag3-S11 | 2.502(5) | Ag19-Ag23^2^ | 2.8723(11) |
| Ag3-S13 | 2.749(5) | Ag19-Ag23 | 2.8723(11) |
| Ag4-Ag5 | 3.024(5) | Ag19-Ag27 | 3.0052(14) |
| Ag4-O3 | 2.45(3) | Ag19-S8 | 2.466(4) |
| Ag4-S11 | 2.782(5) | Ag19-S8^2^ | 2.466(4) |
| Ag4-S13 | 2.797(6) | Ag20-Ag21 | 2.8407(9) |
| Ag4-S14 | 2.497(5) | Ag20-Ag21^2^ | 2.8406(9) |
| Ag5-O4 | 2.46(3) | Ag20-Ag22 | 2.9857(9) |
| Ag5-S11 | 2.368(6) | Ag20-Ag22^2^ | 2.9857(9) |
| Ag5-S17 | 2.386(6) | Ag20-Ag23^2^ | 2.8472(10) |
| Ag6-Ag7 | 2.808(12) | Ag20-Ag23 | 2.8472(10) |
| Ag6-Ag15 | 2.790(7) | Ag20-Ag24^3^ | 2.8476(13) |
| Ag6-O1 | 2.32(6) | Ag20-Ag26^1^ | 2.9838(11) |
| Ag6-S15 | 2.488(9) | Ag20-Ag26^3^ | 2.9838(11) |
| Ag6-S17 | 2.745(11) | Ag20-O17 | 2.334(11) |
| Ag7-Ag18 | 3.134(4) | Ag21-Ag21^1^ | 2.8312(12) |
| Ag7-O2 | 2.43(6) | Ag21-Ag22 | 2.8847(10) |
| Ag7-S9 | 2.423(5) | Ag21-Ag23 | 2.7482(9) |
| Ag7-S12 | 2.359(5) | Ag21-Ag23^1^ | 2.8314(9) |
| Ag8-O5^1^ | 2.27(7) | Ag21-Ag24^3^ | 2.7527(9) |
| Ag8-S13 | 2.420(5) | Ag21-Ag25 | 2.7699(10) |
| Ag8-S16 | 2.434(5) | Ag21-Ag25^1^ | 2.8243(10) |
| Ag9-O11 | 2.275(12) | Ag21-Ag26^1^ | 2.8941(10) |
| Ag9-S13^1^ | 2.382(5) | Ag21-Ag28^1^ | 2.7736(9) |
| Ag9-S16 | 2.479(6) | Ag22-Ag23 | 2.8439(10) |
| Ag10-Ag13^1^ | 3.101(8) | Ag22-Ag25 | 3.0090(10) |
| Ag10-S2 | 2.550(6) | Ag22-S4^1^ | 2.455(3) |
| Ag10-S14^1^ | 2.841(10) | Ag22-S8 | 2.457(3) |
| Ag10-S16^1^ | 2.259(9) | Ag23-Ag23^2^ | 2.7577(15) |
| Ag11-O12 | 2.359(7) | Ag23-Ag24 | 2.8210(10) |
| Ag11-S2 | 2.415(4) | Ag23-Ag25 | 2.7752(11) |
| Ag11-S5 | 2.451(4) | Ag23-Ag27 | 2.7588(10) |
| Ag12-Ag13 | 2.921(6) | Ag23-Ag28 | 2.8225(9) |
| Ag12-Ag15 | 3.373(4) | Ag24-Ag26^2^ | 2.8666(11) |
| Ag12-O10 | 2.257(13) | Ag24-Ag26 | 2.8665(11) |
| Ag12-S14 | 2.491(6) | Ag24-Ag27 | 2.8266(13) |
| Ag12-S17 | 2.345(6) | Ag24-Ag28 | 2.7610(9) |
| Ag13-O9 | 2.441(9) | Ag24-Ag28^2^ | 2.7608(9) |
| Ag13-S2^1^ | 2.575(5) | Ag25-Ag25^1^ | 3.1187(16) |
| Ag13-S10 | 2.547(6) | Ag25-Ag28 | 3.1460(11) |
| Ag13-S14 | 2.942(7) | Ag25-O9 | 2.336(9) |
| Ag14-Ag15 | 3.327(2) | Ag25-O12^1^ | 2.346(9) |
| Ag14-O13 | 2.356(7) | Ag26-Ag26^2^ | 3.349(2) |
| Ag14-S6 | 2.437(2) | Ag26-Ag28 | 3.0078(10) |
| Ag14-S8 | 2.961(4) | Ag26-S4 | 2.459(4) |
| Ag14-S10 | 2.472(4) | Ag26-S7 | 2.475(4) |
| Ag15-O16 | 2.472(9) | Ag27-Ag28 | 3.1403(10) |
| Ag15-S10 | 2.634(4) | Ag27-Ag28^2^ | 3.1401(10) |
| Ag15-S12 | 2.439(5) | Ag27-O13^2^ | 2.326(8) |
| Ag15-S17 | 2.581(6) | Ag27-O13 | 2.326(8) |
| Ag16-S6 | 2.537(6) | Ag28-O8 | 2.304(10) |
| Ag16-S12 | 2.541(5) | Ag28-O14 | 2.317(7) |
| O14-Ag1-S1 | 106.9(2) | S2-Ag11-S5 | 148.21(14) |
| O14-Ag1-S3 | 97.0(2) | O10-Ag12-S14 | 105.1(4) |
| S3-Ag1-S1 | 150.20(13) | O10-Ag12-S17 | 109.5(4) |
| O8-Ag2-S3 | 94.8(2) | S17-Ag12-S14 | 141.4(2) |
| O8-Ag2-S4 | 94.3(2) | O9-Ag13-S21 | 92.2(3) |
| O8-Ag2-S5 | 111.5(2) | O9-Ag13-S10 | 104.0(3) |
| S3-Ag2-S4 | 105.78(13) | O9-Ag13-S14 | 103.7(3) |
| S3-Ag2-S5 | 145.15(12) | S21-Ag13-S14 | 104.25(19) |
| S5-Ag2-S4 | 94.90(12) | S10-Ag13-S21 | 131.9(2) |
| O7-Ag3-S11 | 95.7(3) | S10-Ag13-S14 | 114.9(2) |
| O7-Ag3-S13 | 90.7(3) | O13-Ag14-S6 | 97.4(2) |
| S5-Ag3-O7 | 103.5(2) | O13-Ag14-S8 | 96.07(18) |
| S5-Ag3-S11 | 136.90(16) | O13-Ag14-S10 | 107.7(2) |
| S5-Ag3-S13 | 104.87(16) | S6-Ag14-S8 | 101.26(16) |
| S11-Ag3-S13 | 113.21(17) | S6-Ag14-S10 | 147.90(14) |
| O3-Ag4-S11 | 108.2(6) | S10-Ag14-S8 | 95.74(13) |
| O3-Ag4-S13 | 103.4(7) | O16-Ag15-S10 | 96.2(2) |
| O3-Ag4-S14 | 116.7(6) | O16-Ag15-S17 | 94.4(3) |
| S11-Ag4-S13 | 103.69(16) | S12-Ag15-O16 | 103.2(3) |
| S14-Ag4-S11 | 119.86(19) | S12-Ag15-S10 | 126.14(15) |
| S14-Ag4-S13 | 102.54(17) | S12-Ag15-S17 | 128.23(18) |
| S11-Ag5-O4 | 112.9(7) | S17-Ag15-S10 | 99.01(18) |
| S11-Ag5-S17 | 132.4(2) | S6-Ag16-S12^2^ | 117.97(10) |
| S17-Ag5-O4 | 111.4(6) | S6-Ag16-S12 | 117.97(10) |
| O1-Ag6-S15 | 119.1(16) | S12^2^-Ag16-S12 | 118.2(2) |
| O1-Ag6-S17 | 90.0(13) | S11-Ag17-S3 | 115.50(15) |
| S15-Ag6-S17 | 111.1(4) | S11-Ag17-S15 | 125.05(16) |
| S9-Ag7-O2 | 119.4(15) | S15-Ag17-S3 | 112.63(15) |
| S12-Ag7-O2 | 112.2(15) | O15-Ag18-S1 | 97.9(2) |
| S12-Ag7-S9 | 128.4(3) | O15-Ag18-S9 | 95.2(3) |
| O5^1^-Ag8-S13 | 117.9(19) | S9-Ag18-S1 | 100.60(11) |
| O5^1^-Ag8-S16 | 114(2) | S15-Ag18-O15 | 102.0(3) |
| S13-Ag8-S16 | 127.4(2) | S15-Ag18-S1 | 124.28(16) |
| O11-Ag9-S13^1^ | 108.0(4) | S15-Ag18-S9 | 128.23(15) |
| O11-Ag9-S16 | 107.4(4) | S8-Ag19-S8^2^ | 139.44(16) |
| S13^1^-Ag9-S16 | 142.5(2) | S41-Ag22-S8 | 143.07(11) |
| S2-Ag10-S14^1^ | 107.9(3) | O9-Ag25-O12^1^ | 96.9(3) |
| S16^1^-Ag10-S2 | 128.6(4) | S4-Ag26-S7 | 144.21(11) |
| S16^1^-Ag10-S14^1^ | 116.0(3) | O13-Ag27-O13^2^ | 98.6(4) |
| O12-Ag11-S2 | 100.5(2) | O8-Ag28-O14 | 97.4(3) |
| O12-Ag11-S5 | 107.9(2) |  |  |
| Symmetry codes:(1) 1-*x*, +*y*, 1-*z*; (2) +*x*, 1-*y*, +*z*; (3) 1-*x*, 1-*y*, 1-*z* | | | |

**Reference:**

1. Tang, K. L.; Xie, X. J.; Zhao, L.; Zhang, Y. H.; Jin, X. L. *Eur. J. Inorg. Chem.* **2004**, *2004*, 78-85.
2. Iki, N.; Kabuto, C.; Fukushima, T.; Kumagai, H.; Takeya, H.; Miyanari, S.; Miyashi, T.; Miyano, S. *Tetrahedron*. **2000**, *56*, 1437-1443.
3. Rigaku Oxford Diffraction. *CrysAlis^Pro^ Software system, version 1.171.40.25a*, Rigaku Corporation: Oxford, UK, **2018**.
4. Palatinus, L.; Chapuis, G. *J. Appl. Crystallogr.* **2007**, *40*, 786-790.
5. Sheldrick, G. M. *Acta. Crystallogr. Sect*. *C*. **2015**, *71*, 3-8.
6. Dolomanov, O. V., Bourhis, L. J., Gildea, R. J., Howard, J. A. K.; Puschmann, H. *J. Appl. Crystallogr*. **2009**, *42*, 339-341.
7. Spek, A.L. *Acta. Crystallogr. Sect. D.* **2009**, *65*, 148-155.
8. Perdew, J. P. *Phys. Rev. B* **1986**, *33*, 8822–8824.
9. Becke, A. D. *Phys. Rev. A* **1988**, *38*, 3098-3100.
10. Rüger, R.; Van Lenthe, E.; Heine, T.; Visscher, L. *J. Chem. Phys.* **2016**, *144*, 184103.
11. te Velde, G.; Bickelhaupt, F. M.; Baerends, E. J.; Fonseca Guerra, C.; van Gisbergen, S. J. A.; Snijders, J. G.; Ziegler, T. *J. Comput. Chem.* **2001**, *22*, 931-967.
12. Klamt, A.; Schüürmann, G. *J Chem Soc Perkin Trans 2* **1993**, *5*, 799-805.
13. Klamt, A. *J. Phys. Chem.* **1995**, *99*, 2224-2235.
14. Klamt, A.; Jonas, V. *J. Chem. Phys.* **1996**, *105*, 9972-9981.
15. Margiolaki, I.; Margadonna, S.; Prassides, K.; Hansen, T.; Ishii, K.; Suematsu, H., Magnetic structure of the europium fulleride ferromagnet Eu_6_C_60_. *J. Am. Chem. Soc.* 2002, *124*, 11288-11289.
16. Kroto, H. W.; Heath, J. R.; Obrien, S. C.; Curl, R. F.; Smalley, R. E., C_60_: Buckminsterfullerene. *Nature* 1985, *318*, 162-163.
17. Xu, Y.-H.; Tian, W.-J.; Munoz-Castro, A.; Frenking, G.; Sun, Z.-M., An all-metal fullerene: [K@Au_12_Sb_20_]^5-^. *Science*, 2023, *382*, 840-843.
18. Fang, L.; Fan, W.; Bian, G.; Wang, R.; You, Q.; Gu, W.; Xia, N.; Liao, L.; Li, J.; Deng, H.; Yan, N.; Wu, Z. *Angew. Chem. Int. Ed.* 2023, e202305604.
19. Yang, G.; Mu, X.; Pan, X.; Tang, Y.; Yao, Q.; Wang, Y.; Jiang, F.; Du, F.; Xie, J.; Zhou, X.; Yuan, X. *Chem. Sci.* 2023, *14*, 4308-4318.
20. Zhang, X.; Wang, Z.; Qian, S.; Liu, N.; Sui, L.; Yuan, X., *Nanoscale* 2020, *12*, 6449-6455.
21. Jin, F.; Dong, H.; Zhao, Y.; Zhuang, S.; Liao, L.; Yan, N.; Gu, W.; Zha, J.; Yuan, J.; Li, J.; Deng, H.; Gan, Z.; Yang, J.; Wu, Z., *Acta Chim. Sinica* 2020, *78*, 407-411.
22. Wang, H.-H.; Wei, J.; Bigdeli, F.; Rouhani, F.; Su, H.-F.; Wang, L.-X.; Kahlal, S.; Halet, J.-F.; Saillard, J.-Y.; Morsali, A.; Liu, K.-G., *Nanoscale* 2023, *15*, 8245-8254.
23. Bian, K.; Zhang, X.; Liu, K.; Yin, T.; Liu, H.; Niu, K.; Cao, W.; Gao, D., *ACS Sustainable Chem. Eng.* 2018, *6*, 7574-7588.
24. Zhang, J.; Wang, J.; Fan, G.; Zhang, B.; Ma, G.; Xiao, H.; Wang, L., *Polymers* 2022, *14*, 2787.
25. Liu, Y.; Ai, K.; Liu, J.; Deng, M.; He, Y.; Lu, L., *Adv. Mater.* 2013, *25*, 1353-1359.
26. Wang, Z.; Zhu, Y.-J.; Han, B.-L.; Li, Y.-Z.; Tung, C.-H.; Sun, D., *Nat. Commun.* 2023, *14*, 5295.
27. Wang, Z.; Zhu, Y.-J.; Ahlstedt, O.; Konstantinou, K.; Akola, J.; Tung, C.-H.; Alkan, F.; Sun, D. *Angew Chem Int Ed* 2023, *63*, e202314515.
28. Wang, Z.; Zhao, H.; Li, Y.-Z.; Zhang, C.; Gupta, R. K.; Tung, C.-H.; Sun, D. *Nano Lett* 2023, *24*, 458-465.
29. Wang, Z.; Su, H. F.; Kurmoo, M.; Tung, C. H.; Sun, D.; Zheng, L. S., *Nat. Commun.* 2018, *9*, 2094.
30. Wang, Z.; Yang, F. L.; Yang, Y.; Liu, Q. Y.; Sun, D., *Chem. Commun.* 2019, *55*, 10296-10299.
31. Wang, Z.; Qu, Q.-P.; Su, H.-F.; Huang, P.; Gupta, R. K.; Liu, Q.-Y.; Tung, C.-H.; Sun, D.; Zheng, L.-S., *Sci. China Chem.* 2019, *63*, 16-20.
32. Wang, Z.; Su, H.-F.; Zhuang, G.-L.; Kurmoo, M.; Tung, C.-H.; Sun, D.; Zheng, L.-S., *CCS Chem.* 2020, *2*, 663-672.
33. Liu, J. W.; Wang, Z.; Chai, Y. M.; Kurmoo, M.; Zhao, Q. Q.; Wang, X. P.; Tung, C. H.; Sun, D., *Angew. Chem. Int. Ed.* 2019, *58*, 6276-6279.
34. Su, Y. M.; Wang, Z.; Zhuang, G. L.; Zhao, Q. Q.; Wang, X. P.; Tung, C. H.; Sun, D., *Chem. Sci.* 2019, *10*, 564-568.
35. Wang, Z.; Sun, H. T.; Kurmoo, M.; Liu, Q. Y.; Zhuang, G. L.; Zhao, Q. Q.; Wang, X. P.; Tung, C. H.; Sun, D., *Chem. Sci.* 2019, *10*, 4862-4867.
36. Su, Y.-M.; Ji, B.-Q.; Wang, Z.; Zhang, S.-S.; Feng, L.; Gao, Z.-Y.; Li, Y.-W.; Tung, C.-H.; Sun, D.; Zheng, L.-S., *Sci. China Chem.* 2021, *64*, 1482-1486.
